# Room temperature design of Ce(iv)-MOFs: from photocatalytic HER and OER to overall water splitting under simulated sunlight irradiation†

**DOI:** 10.1039/d2sc05161c

**Published:** 2023-02-24

**Authors:** Shan Dai, Eva Montero-Lanzuela, Antoine Tissot, Herme G. Baldoví, Hermenegildo García, Sergio Navalón, Christian Serre

**Affiliations:** a Institut des Matériaux Poreux de Paris, Ecole Normale Supérieure, ESPCI Paris, CNRS, PSL University 75005 Paris France antoine.tissot@ens.psl.eu christian.serre@ens.psl.eu; b Departamento de Química, Universitat Politècnica de València C/Camino de Vera, s/n 46022 Valencia Spain sernaol@doctor.upv.es; c Instituto de Tecnología Química (CSIC-ITQ) Av de Los Naranjos, s/n 46022 Valencia Spain

## Abstract

The development of MOF-based efficient and reusable catalysts for hydrogen production under simulated sunlight irradiation, especially through overall water splitting, remains challenging. This is mainly due to either the inappropriate optical features or poor chemical stability of the given MOFs. Room temperature synthesis (RTS) of tetravalent MOFs is a promising strategy to design robust MOFs and their related (nano)composites. By employing these mild conditions, herein, we report for the first time that RTS leads to the efficient formation of highly redox active Ce(iv)-MOFs that are inaccessible at elevated temperatures. Consequently, not only highly crystalline Ce-UiO-66-NH_2_ is synthesized, but also many other derivatives and topologies (8 and 6-connected phases) without compromise in space-time yield. Their photocatalytic HER and OER activities under simulated sunlight irradiation are in good agreement with their energy level band diagrams: Ce-UiO-66-NH_2_ and Ce-UiO-66-NO_2_ are the most active photocatalysts for the HER and OER, respectively, with a higher activity than other metal-based UiO-type MOFs. Combining Ce-UiO-66-NH_2_ with supported Pt NPs results finally in one of the most active and reusable photocatalysts for overall water splitting into H_2_ and O_2_ under simulated sunlight irradiation, due to its efficient photoinduced charge separation evidenced by laser flash photolysis and photoluminescence spectroscopies.

## Abstract 2

A new synthetic approach is reported to synthesize redox-active Ce(iv) MOFs at room temperature for efficient and reusable photo-induced overall water splitting.

## Graphical abstract

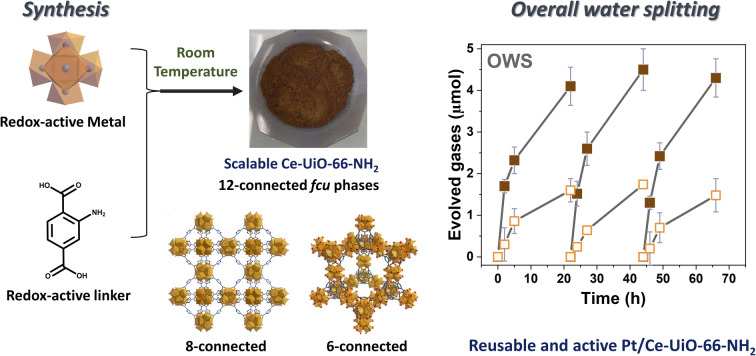

## Introduction

The ever-increasing energy crisis and its environmental impact are strong incentives to look for alternative renewable energy sources that neither rely on the consumption of fossil fuels nor emit carbon dioxide.^1,2^ Solar-driven water splitting is a very promising technology to obtain green hydrogen to meet the carbon neutrality objectives.^3^ Over the years inorganic semiconductors have been intensively employed as photocatalysts for water splitting.^4^ Recently, strong motivation has emerged in the development of efficient hybrid inorganic–organic catalysts. Similar to alloying, organic ligand coordination can modify flexibly the electronic state of inorganic moieties, making them not only more reactive but also more tailorable in figuring out the water splitting.

Porous coordination polymers or metal–organic frameworks (MOFs), are crystalline hybrid materials that consist of metal oxoclusters/ions connected by organic ligands in all dimensions.^5–7^ The large number of metals and organic ligands combinations give rise to potential advantages over other porous solids, as one can fully design their structure and composition depending on the desired applications, such as photocatalysis.^8^ However, the vast majority of visible light responsive transition metal(ii) MOFs are poorly water stable, precluding their application in water splitting. Tetravalent-metal-based (*e.g.*, Zr, Hf, Th, Ce) polycarboxylate MOFs are generally very chemically robust due to their strong coordination bonds. Among potential candidates, Ce(iv)-based MOFs are attractive candidates due to their relatively low cost (*e.g.*, CeO_2_, 6 USD/Kilo^9^), high redox activity, and tailorable functions.^10,11^ Before 2015, most studies were focused on developing Ce(iii)-MOFs due to the challenges related to the potential reduction of Ce(iv), that exhibits low-lying 4f orbitals that can easily accept electrons from the organic solvents under solvothermal conditions.^12,13^ Ce(iv)-UiO-66 is a benchmark robust Ce-MOF built with Ce_6_O_4_(OH)_4_^12+^ inorganic oxoclusters and 1,4-benzene-dicarboxylate (BDC) linkers, sharing many similarities with the isostructural Zr(iv)-UiO-66.^14,15^ The presence of redox Ce(iv) in the metal nodes of Ce-UiO-66 mainly differentiates them from Zr(iv) analogs in photo-physical and redox properties.^16–20^

The typical synthesis of tetravalent metals-based MOFs relies on the solvothermal/hydrothermal route,^21^ which in the case of Ce-MOFs is often associated with the partial reduction of Ce(iv), subsequently resulting in either a relatively low product yield or undesired Ce(iii) phases. A few exciting achievements that tentatively follow a room temperature strategy to prepare Ce(iv)-MOFs have been reported very recently.^22^ However, these methods still suffer from strong limitations either due to the use of specific substrates,^23^ highly toxic co-solvents (DMF),^24^ or from a lack of chemical/structural versatility.^25^ In a nut shell, the room temperature synthesis or ‘RTS’ of Ce(iv)-MOFs is not as a mature as the one of Zr(iv)-MOFs that has been recently reported not only to be highly versatile but also associated to a control of the crystal defect engineering and/or paving the way for the encapsulation of fragile compounds through bottle-around-ship strategy.^22^ Therefore, this calls for a similar strategy for Ce-MOFs that should avoid the use of hazardous substances, the use of solvents, minimize the energy input and be applicable to any structure and composition with different photoreactive properties.

In addition, considering their very good chemical stability and redox character, these Ce(iv)-MOFs are interesting candidates for hydrogen production from water splitting. Truhlar *et al.* recently reported a computational investigation on the photo-reactivity of Ce-UiO-66-X (X refers to the functional groups) by using the Kohn–Sham DFT method, predicting the predominant performance of Ce-UiO-66 for the hydrogen (HER) or oxygen (OER) evolution reaction from water among other metal-substituted M-UiO-66 MOFs (M = Zr, Hf, Th, Ti, U and Ce).^12^ In addition, the presence of functional groups in the Ce-UiO-66-X solids may not only allow decreasing their band gap, thus, favoring the solar light absorption, but also enable the tuning of the highest occupied crystal orbital (HOCO) and lowest unoccupied crystal orbital (LUCO) energy levels depending on the photocatalytic reaction in play. Regardless the interest at theoretical level, systematic experimental investigations that ambition to establish a relationship between water splitting performance and the nature of the functional group in Ce-UiO-66-X is also still to be achieved. In addition, as mentioned above, the reducible character of Ce(iv) makes even more challenging its assembly with redox active linkers under typical solvothermal reaction conditions. For instance, 2-amino-1,4-benzenedicarboxylic acid, also denoted here ‘BDC-NH_2_’, is a typical commercially available electron-donating linker associated with a shift of its optical absorption toward the visible range and an enhancement of both the ligand–metal charge transfer (LMCT) and the separation of electrons and holes upon photoexcitation compared to BDC. This topical ligand has so far been combined with almost any type of metals leading to series of amino-functionalized benchmark MOFs such as Zn-MOF-5-NH_2_,^26^ Cr-MIL-101-NH_2_,^27^ Al-MIL-53-NH_2_,^28^ Fe-MIL-88-NH_2_,^29^ Ti-MIL-125-NH_2_,^30^ Zr-UiO-66-NH_2_,^31^ among many others. Concerning Ce-UiO-66-NH_2_, very recent efforts to prepare this phase have been focused on either the use of a solvent-assisted linker exchange (SALE, up to 85% linker exchange) in DMF, where the BDC-NH_2_ could replace the original BDC ligand from the framework,^32^ or on the fabrication of Ce-UiO-66-NH_2_ through the use of pre-formed Ce_6_ oxoclusters in DMF.^33^ Therefore, green easy synthesis of this latter MOF represents still an open challenge.

Herein, we report for the first time a room temperature water-based strategy to produce a series of benchmark Ce(iv)-MOFs with very high space-time yield, including 12-connected Ce-UiO-66-X (X = H, Br, NO_2_, COOH), 8-connected Ce-DUT-67 and 6-connected Ce-MOF-808. This comprises the green *de novo* ambient conditions preparation of the challenging Ce-UiO-66-NH_2_ that exhibits a very narrow band gap of *ca.* 2 eV. Noteworthy, we exploit this new strategy to establish for the first time that Ce-UiO-66-X can be active HER and OER photocatalysts under simulated sunlight irradiation with Ce-UiO-66-NH_2_ and Ce-UiO-66-NO_2_ exhibiting the highest HER and OER activity, respectively, due to their distinct optical features. In addition, we evidence that the deposition of Pt NPs on the Ce-UiO-66-NH_2_ leads to one of the most efficient reusable photocatalysts for overall water splitting (OWS).

## Results and discussion

**Fig. 1:**
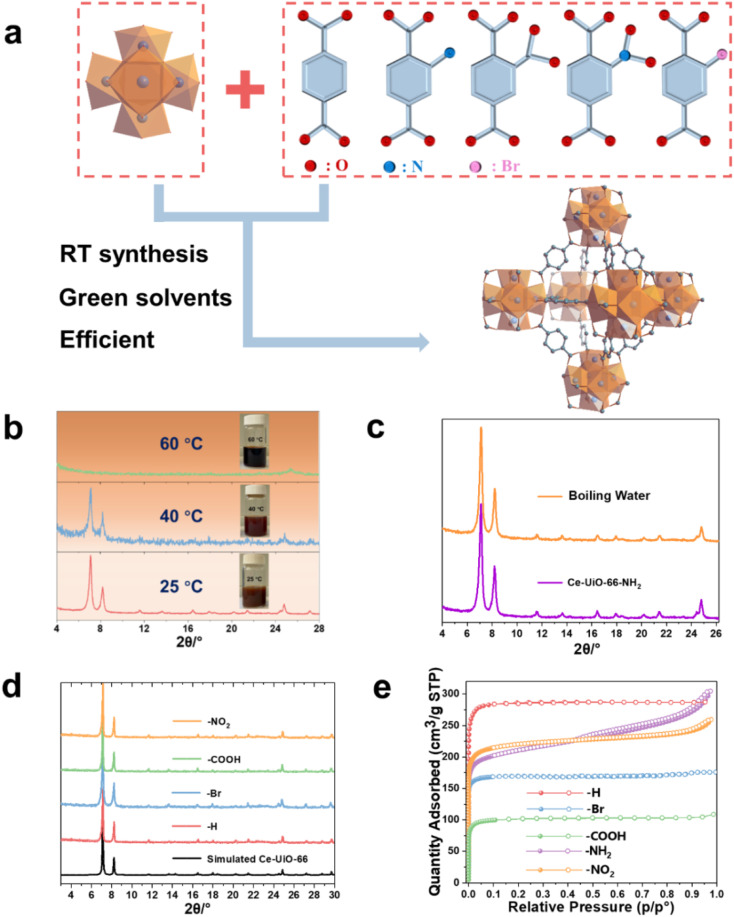
(a) Crystal structures of Zr_6_ oxo-cluster, linkers, and Ce-UiO-66. (b) PXRD (*λ*_Cu_ = 1.5406 Å) patterns of the Ce-UiO-66-NH_2_ synthesized at various temperatures (25 °C, 40 °C, 60 °C), insert image: the photography of the resulting solution. (c) PXRD patterns (*λ*_Cu_ = 1.5406 Å) of the Ce-UiO-66-NH_2_ sample before and after boiling water treatment. (d) PXRD patterns (*λ*_Cu_ = 1.5406 Å) of the RT synthesized Ce-UiO-66-X (X = –H, –NO_2_, –COOH, –Br). (e) 77 K N_2_ adsorption–desorption isotherms of the RT synthesized Ce-UiO-66-X.

The Ce(iv)-UiO-66-X (X = functional groups) is built up by Ce_6_(μ_3_-O)_4_(μ_3_-OH)_4_ oxoclusters that are connected by 1,4 BDC^2−^ or its derivatives, resulting in 3D cubic microporous structures (Fig. 1a). As the auto-reduction of Ce(iv) has also been observed in long-term storage in aqueous conditions without heating,^34,35^ we suspected that developing a room temperature synthetic ‘RTS’ strategy of these Ce-MOFs, similar to the one previously reported for Zr-MOFs, could prevent the strong reducible character of Ce(iv) in solution. As described in the ESI,† we selected the Ce(iv) ammonium nitrate as a low corrosive metal source to be associated first with BDC-NH_2_ in the presence of water, acetic acid and ethanol (that act as co-solvent here) at room temperature. As shown in Fig. 1b, this results in the formation of a brownish solid after 1 h. Powder X-ray diffraction (PXRD) analysis confirms the formation of a highly crystalline Ce-UiO-66-NH_2_. Scanning electron microscopy (SEM) imaging (see Fig. S1†) evidences the formation of *ca.* 80 nm nanocrystals, in agreement with the slight broadening of the Bragg peaks. Notably, a slight increase of the synthesis temperature (40 °C) leads to a much less crystalline solid, while further increasing the temperature up to 60 °C results in a clear dark solution without any solid. This highlights that only near ambient conditions enables the successful nucleation of Ce-UiO-66-NH_2_, likely due to the reduction of Ce(iv) at higher temperature, probably due to slow reduction kinetics at room temperature compatible with the kinetics of MOF crystallization. The alternative use of the same quantity of stronger modulator (formic acid) at room temperature doesn't result in lower product yield regardless of the prolonged synthesis duration (to 2 h), which again evidences the formation of Ce-UiO-66-NH_2_ is more temperature dependent. Despite these promising results, the relatively low product yield (42%) is likely to be due to a partial reduction of Ce(iv) even at RT, however without the formation of undesired Ce(iii)-MOFs. This, however, can be overcome by using an excess of Ce(iv) salt (1.7 eq.), giving rise to a significantly higher product yield (76%) based on the ligand. To the best of our knowledge, this is the first example of room temperature synthesis to form a Ce(iv)-MOF based on a highly redox active ligand. Considering the previous failures for the aqueous synthesis of Ce-UiO-66-NH_2_ at elevated temperature, we subsequently investigated the chemical stability of the as-synthesized Ce-UiO-66-NH_2_ by soaking this MOF in boiling water for 24 h. Surprisingly, the PXRD pattern (Fig. 1b) supports the hydrolytic robustness of Ce-UiO-66-NH_2_. As Ce(iv) cations are fully exposed to solvent molecules present in solution that are in competition with oxygen species from the Ce_6_(μ_3_-O)_4_(μ_3_-OH)_4_ node, one can assume that the very good chemical stability might come from a stabilization of Ce(iv) sites by these O/OH species. To be noted, these results are consistent with a previous study, where Tang *et al.* reported that the use of pre-synthesized Ce_6_ oxoclusters (Ce_6_O_4_(OH)_4_(NH_3_–CH_2_COO)_8_(NO_3_)_4_(H_2_O)_6_Cl_8_·8H_2_O) could form Ce-UiO-66-NH_2_ at 100 °C.^33^

Subsequently, we have extended this room temperature synthesis to other UiO-66 derivatives, including Ce-UiO-66-X (X = H, Br, NO_2_, COOH). Due to the poor solubility of BDC in the sole presence of water, ethanol is used as a co-solvent in this case. After only 1–2 h of stirring at room temperature, the products are isolated and washed. The PXRD analysis (Fig. 1d) reveals that the expected frameworks were obtained, demonstrating the versatility of this strategy to prepare a large number of Ce-UiO-66-X with high crystallinity (SEM images, Fig. S1†). However, when heating the reaction solution to 100 °C, lower product yield (*ca.* 69%) is obtained, most likely due to the reduction of Ce(iv). The Fourier transform infrared spectroscopy (FTIR) spectra in Fig. S2 and Table S1† evidence the presence of typical vibrations from coordinated terephthalate and functional groups, further indicating the formation of the corresponding structures. The N_2_ adsorption–desorption isotherms at 77 K are conducted to investigate the porosity of the prepared Ce-UiO-66-X and are shown in Fig. 1e. All the prepared Ce-UiO-66-X display archetypal type-I isotherms, in agreement with the microporous character of these materials. The specific surface area, pore size distribution and micropore volume are calculated from Brunauer–Emmett–Teller (BET) analyses of the adsorption data (shown in Table 1). These values are in agreement with the values reported from the conventional synthesis in DMF, which further exemplifies the high quality of our synthesized MOFs.^15^ In comparison, the previously reported successful preparation of Ce-UiO-66-NH_2_ through solvothermal synthesis using pre-synthesized Ce_6_ oxoclusters led to a much lower BET surface area of 445 m^2^ g^−1^*vs.* 820 m^2^ g^−1^ in this work, due to the relatively poor crystallinity of the solid. The variations within surface area values of the functionalized Ce-UiO-66 can typically be attributed to the introduced functional groups, which increase the density of porous solids, as well as their slightly distinct defect content (Fig. S4 and S5,† summarized in Table S2†).

**Table 1:** The room-temperature synthesized Ce-MOFs in this work

Entry	Materials	Solvents	Modulator	Connectivity (linker, metal)	BET surface area (m^2^ g^−1^)	Pore volume (cm^3^ g^−1^)	STY (kg m^−3^ per day)
1	Ce-UiO-66	H_2_O, ethanol	Acetic acid	(2, 12)	1182	0.44	425
2	Ce-UiO-66-NH_2_	H_2_O, ethanol	Acetic acid	(2, 12)	819	0.38	311
3	Ce-UiO-66-NO_2_	H_2_O	Acetic acid	(2, 12)	878	0.36	886
4	Ce-UiO-66-Br	H_2_O	Acetic acid	(2, 12)	698	0.26	923
5	Ce-UiO-66-COOH	H_2_O	Acetic acid	(2, 12)	418	0.16	839
6	Ce-DUT-67 (PDA)	H_2_O	Acetic acid	(2, 8)	495	0.22	435
7	Ce-MOF-808	H_2_O	Formic acid	(3, 6)	1802	0.65	769

From an industrial production point of view, minimizing the energy input and maximizing the space-time yield (STY) is of critical importance. In this regard, RT synthesis is often associated to low-moderate STY due to the slower crystal formation. While benefiting from the short preparation time (1–2 h) in our case, the space-time-yield (STY) for all these Ce-UiO-66-X reaches high values of 351–900 kg m^−3^ per day (summarized in Table 1), on the whole comparable to that of hydro/solvothermal strategies of many benchmark MOFs^36,37^

**Fig. 2:**
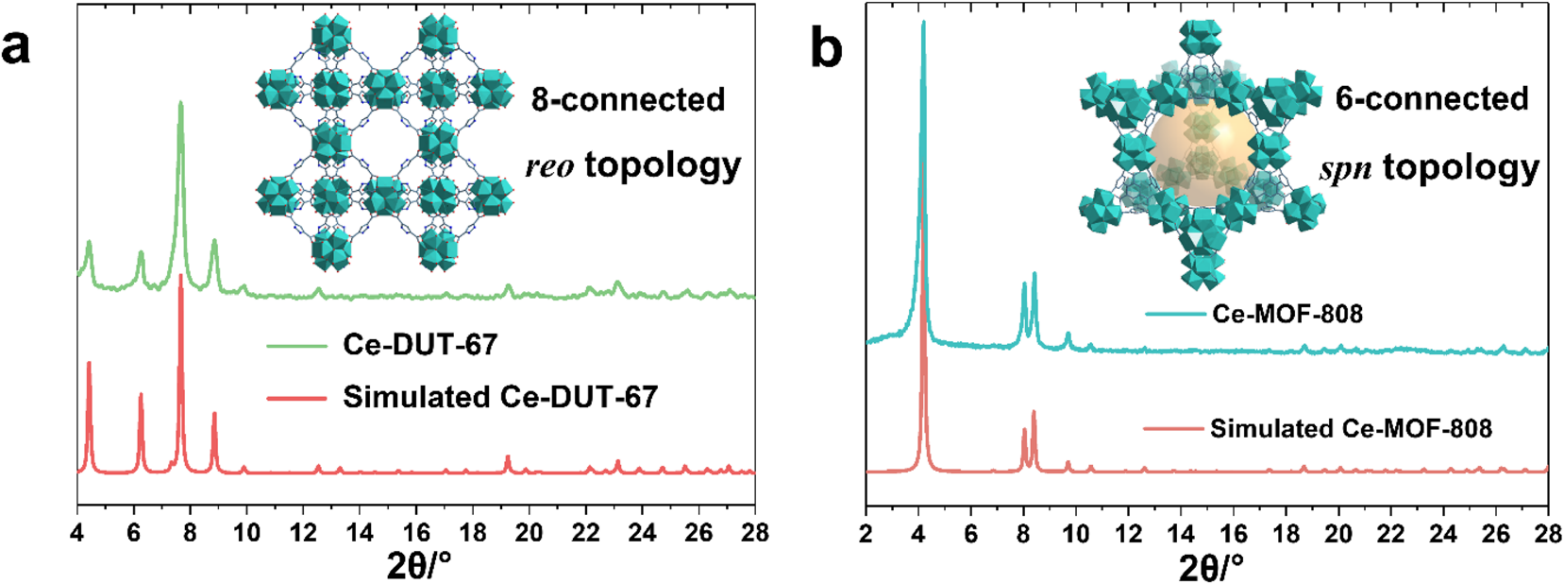
PXRD patterns (*λ*_Cu_ = 1.5406 Å) of synthesized (a) 8-connected *reo* Ce-DUT-67(PDA) and (b) 6-connected *spn* Ce-MOF-808 in comparison to simulated patterns, insert images: structure schemes of the corresponding MOFs.

The large library of available organic linkers enables to construct MOFs with distinct dimensionality and porosity. For example, using 1,3,5-benzenetricarboxylic acid (BTC) and 1*H*-pyrazole-3,5-dicarboxylic acid (PDA), common tritopic and ditopic ligands (angled coordination sites) respectively, while adjusting the ligand stoichiometry, lead to highly crystalline 6-connected Ce-MOF-808 and 8-connected DUT-67(PDA) Ce_6_ oxocluster based MOFs, with distinct porous features and chemical affinities (Fig. 2a, b). Notably, the synthesis of MOF-808 needs the presence of one equivalent of formic acid instead of acetic acid, otherwise only amorphous products can be obtained. This is probably due to the lower p*K*_a_ of BTC, which requires more acidic modulator (p*K*_a,formic acid_ = 3.7 *vs.* p*K*_a,acetic acid_ = 4.7) to effectively control the crystallization kinetics.

**Fig. 3:**
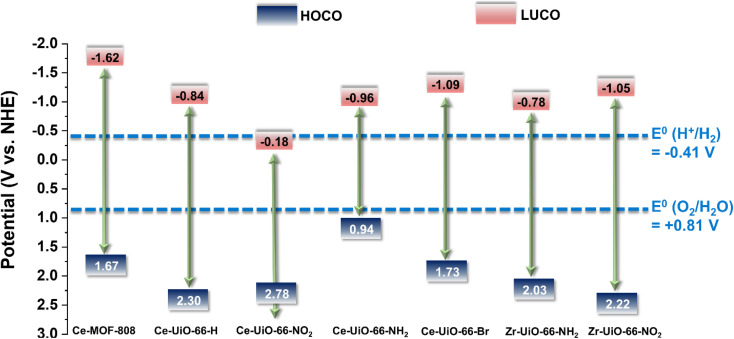
Energy level diagram *vs.* NHE and vacuum of substituted Ce-UiO-66-X and MOF-808(Ce) materials as well as Zr-UiO-66-NH_2_ and Zr-UiO-66-NO_2_ taken as reference compounds.^41^ The figure also indicates the required potential for the HER or OER *vs.* NHE at pH 7.

To effectively use the visible light for water splitting, the typical band gap of a photocatalyst should lie from 2.0 to 3.0 eV.^38^ According to the previous theoretical and experimental reports,^12,33^ as well as our UV-vis absorption spectra (Fig. S6,† summarized in Table S3†), all Ce-UiO-66-X possess suitable band gaps (Fig. 3). To have efficient visible-light-driven photocatalytic water splitting, using the materials with the lowest band gap while fulfilling the thermodynamic requirements of the reaction is appealing due to the potential maximized light harvest. Hence, in this regard, Ce-UiO-66-NH_2_ might be more competitive among the Ce-based MOFs. As the targeted application is water splitting, we subsequently performed H_2_O sorption isotherms at 298 K (Fig. S7†) as the difference in Ce-UiO-66 derivatives affinity to H_2_O may affect the photocatalytic water splitting efficiency due to the modulation of the host–guest interactions. All the functionalized materials show high water uptake and a clear step in the 0.18 to 0.26 *P*/*P*_0_ range. The step pressure follows the order Ce-UiO-66-NH_2_ > Ce-UiO-66-NO_2_ > Ce-UiO-66-Br, indicating the higher surface hydrophilicity of Ce-UiO-66-NH_2_ compared to Ce-UiO-66-Br.

The series of the MOFs under study were characterized by XPS. Fig. S8–S13† collect the survey and high resolution XP spectra of Ce-UiO-66-X (X = H, NO_2_, NH_2_, Br) and Ce-MOF-808. The C 1s spectra of the MOFs are characterized by a main band centered at 284.4 eV due to the sp^2^ C carbon atoms of terephthalate or trimesate. The C 1s spectra of Ce-UiO-66-NH_2_ has an additional component at 285.5 eV due to the C–NH_2_, while those of Ce-UiO-66-NO_2_ and Ce-UiO-66-Br exhibit a component at 286.0 eV due either to C–NO_2_ or to C–Br carbon atoms. The high resolution XPS O 1s spectra of the MOFs have components associated with the presence of oxygen atoms in the carboxylate (532.0 eV) and NO_2_ group (532.5 eV) together with those oxygen atoms present in the cerium or zirconium oxo clusters (529.0 eV). The XPS N 1s peaks of Ce-UiO-66-NH_2_ or Ce-UiO-66-NO_2_ shows the characteristic signal associated with the amino (399.0 eV) and nitro (405.5 eV) groups. The Ce 3d spectra of the Ce-UiO-66-X series and Ce-MOF-808 shows the presence of three main contributions due of Ce(iv) 3d_5/2_ at 885.0 eV and Ce(iv) 3d_3/2_ at 900 and 918 eV.

With the motivation of precedent studies highlighting the potential use of Ce-MOFs as photocatalysts for water splitting,^12,20^ particularly the Ce-UiO-66-X series due to their tunable band alignment diagram, we initially investigated systematically their performance for the independent photocatalytic HER and OER under simulated sunlight irradiation. It is worth mentioning that in comparison with the large number of studies using MOFs as photocatalysts for the HER, the reports describing photocatalytic OER promoted by MOFs are still scarce.^39,40^Fig. 3 shows the experimentally determined energy diagram of Ce-UiO-66-X and Ce-MOF-808 solids as well as those of Zr-UiO-66-NH_2_ and Zr-UiO-66-NO_2_ taken from ref. 41 As commented in the introduction, it is interesting to note the tunability of the frontier orbitals energy of the Ce-UiO-66 photocatalysts by introducing electron donor (*i.e.* NH_2_) or electron withdrawing (*i.e.* NO_2_) functional groups on the terephthalate linker. In principle, Ce-UiO-66-X photocatalysts such as Ce-UiO-66-NH_2_ having more negative LUCO values are expected to be more efficient to promote the HER. Similarly, Ce-UiO-66-X photocatalysts, such as Ce-UiO-66-NO_2_, having more positive HOCO values should favor the OER. Fig. 3 summarizes the energy level diagram of the Ce-UiO-66 samples under study, while ESI† provides details on how these energy values have been determined from XPS valence band maximum measurement (Fig. S13†) and the optical bandgap determination (Fig. S6†). The Ce-UiO-66-X series have been considered as direct bandgap semiconductors based on the relatively minor Stokes shift between absorption (Fig. S6†) and photoluminescence (Fig. S21†).

**Fig. 4:**
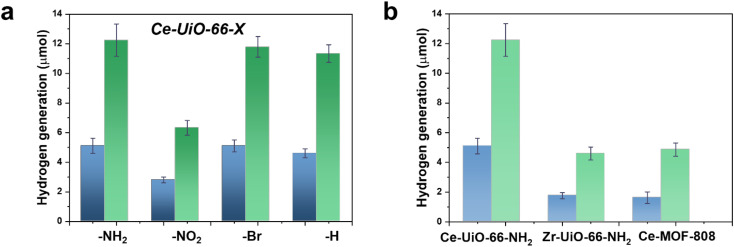
(a) Photocatalytic activity of Ce-UiO-66-X materials for the HER under simulated sunlight irradiation after 1 h (blue bar) or 3 h (green bar). (b) Comparison of the photocatalytic activity of Ce-UiO-66-NH_2_ with Zr-UiO-66-NH_2_ and Ce-MOF-808 after 1 h (blue bar) or 3 h (green bar) simulated sunlight irradiation. Reaction conditions: photocatalyst amount (20 mg), water (16 mL), methanol (4 mL), simulated sunlight irradiation (150 W Hg–Xe lamp equipped with an AM 1.5G filter), 35 °C.

With these comments in mind, we first screened the activity of the Ce-UiO-66-X solids for the HER in the presence of methanol as sacrificial electron donor under simulated sunlight irradiation. As shown in Fig. 4a, the Ce-UiO-66-NH_2_ solid is slightly more active than Ce-UiO-66-Br or Ce-UiO-66 in good agreement with their energy-level diagram. The less active material for HER of the series is Ce-UiO-66-NO_2_ in accordance with its lower LUCO reduction potential. Therefore, the notable influence of terephthalate functionalization on the photocatalytic HER activity can be easily rationalized based on Fig. 3 and the conduction band energy values of each Ce-UiO-66-X material. To put these values into context, additional photocatalytic experiments using Zr-UiO-66-NH_2_ and Ce-MOF-808 as reference materials (see also Fig. 3 for their calculated conduction band energy values) were carried out. The obtained results indicate that the photocatalytic activity of Ce-UiO-66-NH_2_ outperforms that of Zr-UiO-66-NH_2_ and Ce-MOF-808 (Fig. 4b). As theoretically predicted by Truhlar *et al.*, experimental data support that the Ce-UiO-66 based materials are more appropriate as photocatalysts for the HER respect to their other M(IV) analogous (*e.g.*, Zr), probably due to the low-lying Ce^4+^ empty 4f orbitals of Ce-UiO-66-NH_2_ that favors the LMCT mechanism.^12^ In addition, even though Ce-UiO-66-NH_2_ and Zr-UiO-66-NH_2_ have similar ability to promote the HER with LUCO values of –0.96 and −0.78 V, respectively, the smaller band gap of Ce-UiO-66-NH_2_ (1.9 V) respect to the Zr-UiO-66-NH_2_ (2.81 eV) favors HER under simulated sunlight irradiation due to the larger number of photons harvested. Similarly, the smaller band gap and appropriate LUCO values of Ce-UiO-66-NH_2_ respect to Ce-MOF-808 favor the HER efficiency of the former.

Using the best-performing Ce-UiO-66-NH_2_ photocatalyst, a longer 22 h HER run was carried out. The results are presented in Fig. S14 in the ESI.† It was observed that in comparison with the results shown in Fig. 4, H_2_ evolution does not grow linearly at long irradiation times and the amount of H_2_ evolved tend to level off upon irradiation. This result is understandable considering that in batch reactions a stationary H_2_ concentration can be reached due to increasing rate of H_2_ decomposition, as well as the depletion of methanol as sacrificial agent and the generation of reaction by-products from it that act as poison of the active sites.

**Fig. 5:**
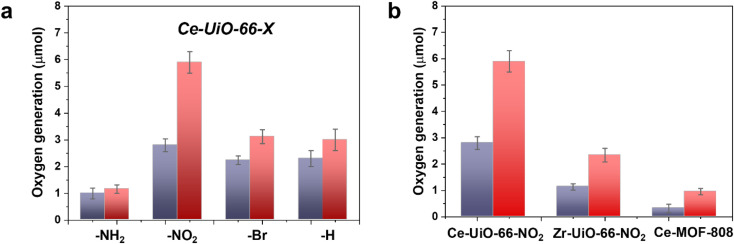
(a) Photocatalytic activity of Ce-UiO-66-X materials for the OER under simulated sunlight irradiation after 1 h (purple bar) or 3 h (red bar). (b) Comparison of the photocatalytic activity of Ce-UiO-66-NH_2_ respect to Zr-UiO-NH_2_ and Ce-MOF-808 after 1 h (purple bar) or 3 h (red bar) simulated sunlight irradiation. Reaction conditions: photocatalyst amount (20 mg), water (20 mL), potassium persulfate (700 mg), simulated sunlight irradiation (150 W Hg–Xe lamp equipped with an AM 1.5G filter), 35 °C.

In a second step, we studied the photocatalytic activity of the Ce-UiO-66-X solids as photocatalysts for the OER in the presence of persulfate as sacrificial agent under simulated sunlight irradiation. In this case, the Ce-UiO-66-NO_2_ outperforms the other Ce-MOFs as OER photocatalyst (Fig. 5a). This can be explained considering that it is the material with the larger overpotential due to its most positive HOCO energy. This should facilitate the H_2_O oxidation, even though Ce-UiO-66-NO_2_ is not the photocatalyst with the smallest band gap under study. In contrast, despite its small band gap, Ce-UiO-66-NH_2_ results in a low photocatalytic activity for the OER, mainly attributable to their inappropriate HOCO energy level alignment respect the O_2_/H_2_O pair redox potential. Therefore, as in the case of HER, the band energy diagram shown in Fig. 3 explains the influence of terephthalate substituent on the photocatalytic OER activity. The photocatalytic activity of the most active Ce-UiO-66-NO_2_ for the OER was also higher than that of Zr-UiO-66-NO_2_ taken as reference photocatalyst (Fig. 5b). Based on previous theoretical studies, it was also proposed that the Ce-based UiO-66s are more appropriate photocatalysts compared to the Zr analogues due to their more favorable LUCO–HOCO overlap facilitating LMCT mechanism.^12^ The higher photocatalytic activity of Ce-UiO-66-NO_2_ respect to the Ce-MOF-808 in Fig. 5b can be attributed to the more appropriate energy level diagram of the former having smaller band gap value and appropriate HOCO energy alignment for the OER.

One general comment regarding Fig. 5 is that O_2_ evolution does not grow linearly with the time in most of the cases and the photocatalytic OER rate at 3 h is lower than expected considering the O_2_ production at 1 h and assuming a linear growth. This indicates that the efficiency of the photocatalytic OER decreases over the time. Among the various possible reasons for photocatalytic activity decrease, the most likely are the different stationary O_2_ concentration depending on the terephthalate substituent, poisoning of the photocatalyst by the decomposition byproducts of the sacrificial electron acceptor and saturation of MOF pores by O_2_ appear to be the most reasonable.

Similarly to the case of HER, also for the best-performing OER photocatalyst a long 22 h run was carried out. The results are presented in Fig. S15 of the ESI.† It was observed that the O_2_ evolution does not grow linearly with the irradiation time and that it tends to level off. This kinetics for batch reaction can reflect the increasing rate of O_2_ decomposition by photogenerated electrons as well as the depletion of the sacrificial electron acceptor and the poisoning effect of by-product generated by it.

**Fig. 6:**
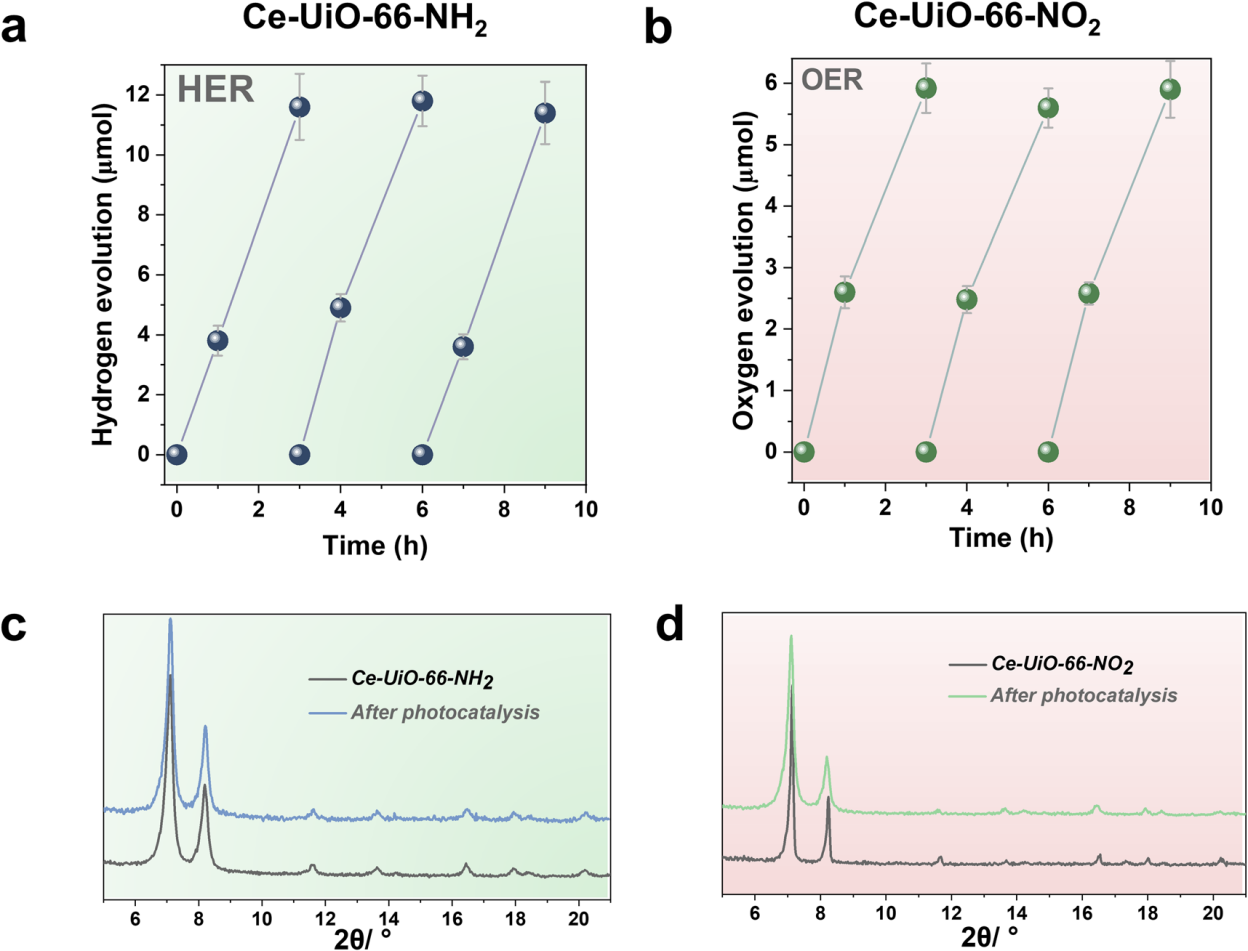
Photocatalytic reusability of Ce-UiO-66-NH_2_ (a) or Ce-UiO-66-NO_2_ (b) for the HER (a) and OER (b), PXRD patterns of Ce-UiO-66-NH_2_ before and after photocatalytic recycling (c) and Ce-UiO-66-NO_2_ before and after photocatalytic recycling (d). Reaction conditions for the HER: photocatalyst amount (20 mg), water (16 mL), methanol (4 mL), simulated sunlight irradiation (150 W Hg–Xe lamp equipped with an AM 1.5G filter), 35 °C. Reaction conditions for the OER: photocatalyst amount (20 mg), water (20 mL), potassium persulfate (700 mg), simulated sunlight irradiation (150 W Hg–Xe lamp equipped with an AM 1.5G filter), 35 °C.

One important issue to be addressed in photocatalysis, especially when using MOF-based materials, is their reusability and stability of the catalyst under reaction conditions. Thus, the most active Ce-UiO-66-NH_2_ and Ce-UiO-66-NO_2_ HER and OER photocatalysts of the series under simulated sunlight irradiation were reused several times. Fig. 6a and b show that the photocatalytic activity of Ce-UiO-66-NH_2_ or Ce-UiO-66-NO_2_ is maintained at least during three consecutive cycles, while the crystallinity of the compounds is maintained according to the PXRD of the three-times used photocatalysts (Fig. 6c and d). However, ICP-AES analysis of the used photocatalysts revealed the occurrence of some metal leaching during the irradiations. The amount of Ce leaching during the photocatalytic HER or OER corresponds, respectively, to about 4 and 6% of the initial Ce amount present in the fresh solids. The higher Ce leaching in the OER respect to the HER may reflect the fact that H_2_O oxidation to O_2_ is thermodynamically a more demanding process respect to the H^+^ reduction to H_2_. For comparison, the stability of the Zr-UiO-66-NH_2_ and Zr-UiO-66-NO_2_ solids were evaluated under similar reaction conditions, finding that these Zr-MOFs also retain their crystallinity according to PXRD after use (Fig. S16–S17†), while the Zr leaching was negligible. Overall, these results manifest that the highest photocatalytic activity of the Ce-based UiO-66 solids is accompanied by a somewhat lower chemical stability respect to the less active, but more stable Zr-based UiO-66 solids.

**Fig. 7:**
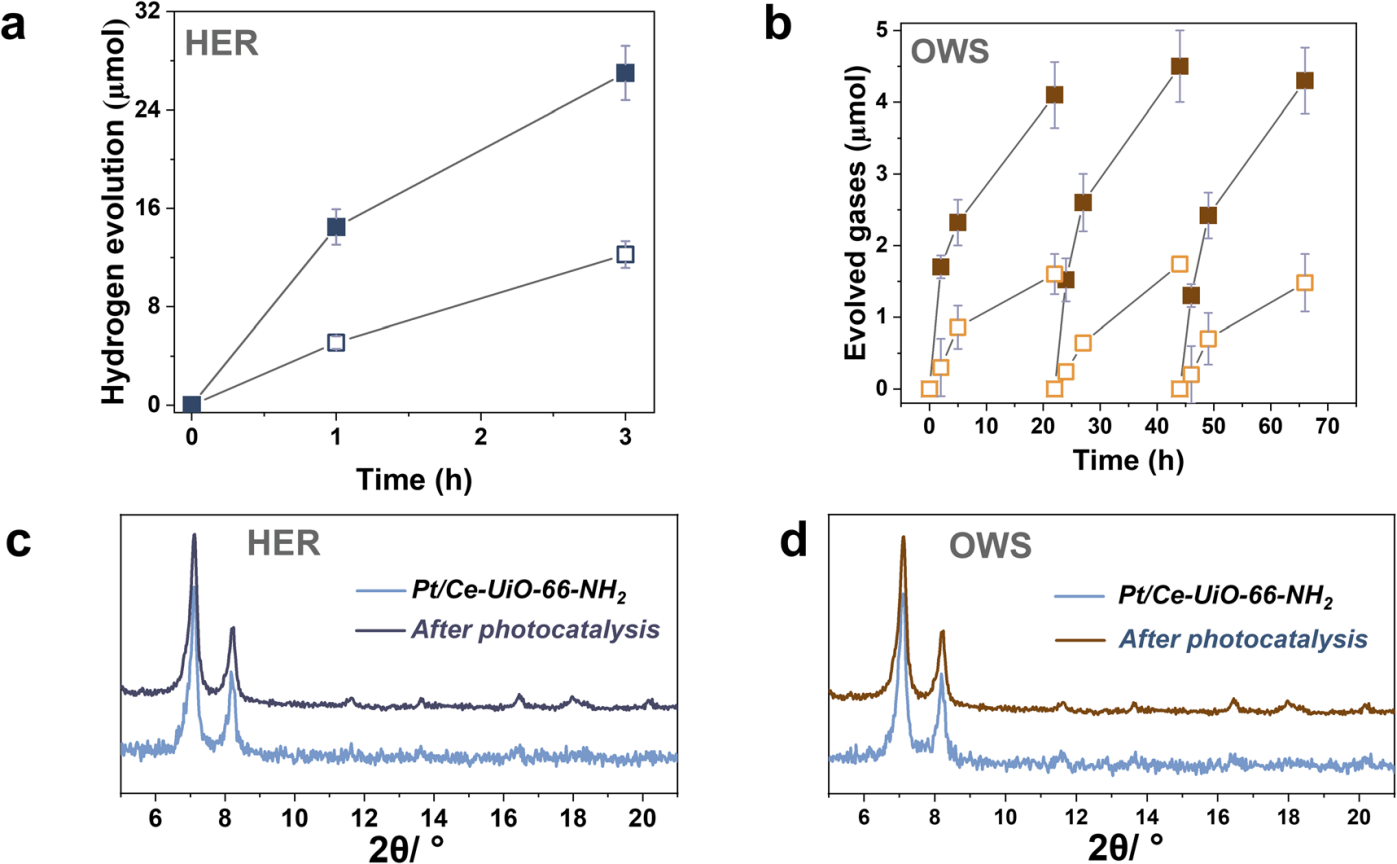
(a) Photocatalytic HER using Ce-UiO-66-NH_2_ (empty square) or Pt/Ce-UiO-66-NH_2_ (filled square) and, (c) the corresponding PXRD pattern of Pt/Ce-UiO-66-NH_2_ before and after catalysis. (b) Photocatalytic overall water splitting (OWS) into H_2_ (filled square) and O_2_ (emptied square) using Pt/Ce-UiO-66-NH_2_ and, (d) the corresponding PXRD patterns of Pt/Ce-UiO-66-NH_2_ before and after photocatalytic recycling. Reaction conditions: photocatalyst amount (20 mg), water (20 mL), simulated sunlight irradiation (150 W Hg–Xe lamp equipped with an AM 1.5G filter), 35 °C.

To further study the photocatalytic HER using the most active Ce-UiO-66-NH_2_ solid, Pt NPs at 1 wt% loading were photodeposited within its framework (labeled as Pt/Ce-UiO-66-NH_2_). Pt NPs are one of the reference and benchmark co-catalyst employed during the photocatalytic HER.^4^ Fig. S18–S19† shows some representative DF-STEM images, Pt particle size distribution and EDX analysis of selected areas. The results indicate the presence of small Pt NPs (1.3 ± 0.6 nm) well distributed through the MOF network (see Fig. S20†). The obtained photocatalytic results indicated that the Ce-UiO-66-NH_2_ loaded with Pt NPs solid exhibits about the double of activity (1200 μmol g^−1^ after 3 h) respect to the parent solid (600 μmol g^−1^ after 3 h) under similar conditions of simulated sunlight irradiation (Fig. 7a). Importantly, the Pt/Ce-UiO-66-NH_2_ photocatalyst retains its crystallinity as evidenced by PXRD (Fig. 7c) and exhibits a significantly lower cerium leaching (0.8 wt% of the initial cerium content in the solid) respect to the parent Ce-UiO-66-NH_2_ solid (4 wt%). In order to obtain some experimental evidences about the higher photocatalytic activity of Pt/Ce-UiO-66-NH_2_ respect to Ce-UiO-66-NH_2_, a photoluminescence study was carried out. Fig. S21† shows that the presence of Pt NPs within the Ce-UiO-66-NH_2_ results in a decrease of PL of about 75% respect to the parent Ce-UiO-66-NH_2_. These results can be interpreted considering that the presence of Pt NPs disfavors charge recombination that is the process responsible for the PL emission.^42^ In fact, in good agreement with theoretical calculations, transient absorption spectroscopy (TAS) measurements using the Pt/Ce-UiO-66-NH_2_ further confirm the occurrence of photoinduced charge separation (Fig. S22 in ESI†).^12^ Specifically, TAS of Pt/Ce-UiO-66-NH_2_ upon irradiation at ligand-centered absorption at 268 nm under Ar atmosphere shows a continuous absorption spectrum from 350 to 700 nm with the same decay profile along the entire spectrum, suggesting that the absorption corresponds to a single species or that if there are several species, they decay by the same mechanism. Decay profiles show two components: one quick and intense and a residual component with longer lifetime (Fig. S23 in ESI†). Interestingly, the use of methanol as electron donor and oxygen as electron quencher results in a spectrum characterized by a notable decrease of TAS intensities between 350 and 600 nm and faster decays in the 350–600 nm range, supporting that this region corresponds predominantly to photogenerated holes. In addition, the presence of methanol also increases the lifetime and intensities above 600 nm, suggesting that there is a contribution of a different species in this spectral region. Additional quenching experiments using molecular O_2_ as electron quencher evidence an intensity increase in the 350–600 nm region that is attributed to the enhancement of photo-generated holes, showing a quenching of the TAS intensities above 600 nm ascribed to the quenching of photo-generated electrons. Furthermore, the use of N_2_O as electron acceptor also results in an intensity increase of signal in most of the spectral window. Therefore, quenching studies are in agreement with the simultaneous photoinduced generation of holes and electrons, the former absorbing preferentially in the 350–600 nm region and being quenched by methanol and electrons absorbing predominantly beyond 600 nm and being quenched by O_2_. Overall, the higher photocatalytic activity and stability of the Pt/Ce-UiO-66-NH_2_ can be attributed to the ability of Pt NPs to both accumulate the photogenerated electrons and act as co-catalyst for the proton reduction to H_2_ in such a way that it increases the stability of the Ce-UiO-66-NH_2_ solid.

The previous HER (Fig. 4) and OER (Fig. 5) data for the Ce-UiO-66-X have shown the remarkable influence of terephathalate substituents on the photocatalytic activity and how the relative photocatalytic activity can be rationalized based on the HOCO and LUCO band energy values shown in Fig. 3. Since OWS is composed of HER and OER semi reactions, terephathalate substituents must influence OWS as well. From HOCO/LUCO energy data of Fig. 3, it can be inferred that Ce-UiO-66-NH_2_ is the most suitable material. Therefore, the photocatalytic activity of Pt/Ce-UiO-66-NH_2_ was further evaluated for the solar-driven photocatalytic overall water splitting (OWS) into H_2_ and O_2_. Fig. 7b shows the temporal H_2_ and O_2_ under simulated sunlight irradiation using Pt/Ce-UiO-66-NH_2_ as photocatalysts in three consecutive reuses, while Table S4 in the ESI† gathers the H_2_/O_2_ molar ratio at each irradiation time for the three runs. As it can be observed in Fig. 7b and, better in the data provided in Table S4,† the amount of evolved O_2_ in the gas phase is below that corresponding to the 0.5 stoichiometry expected for ideal overall water splitting, particularly at shorter irradiation times. This fact has been frequently observed in related precedents of OWS using MOFs as photocatalysts^43–45^ and can be attributed to the operation of various factors having larger contribution at initial reaction times. Among them, the occurrence of 2e^−^ oxidation of H_2_O, building a stationary low concentration of H_2_O_2_ that subsequently decomposes to O_2_ (the 4e^−^ oxidation product) and saturation of deareated H_2_O by O_2_, much more water-soluble than H_2_, are two major processes to be considered to justify the lower-than-the-stoichiometry O_2_ amount measured in the gas phase. The three-times used Pt/Ce-UiO-66-NH_2_ photocatalyst retains its crystallinity as revealed by PXRD (Fig. 7d). ICP-OES analysis further confirmed the stability of the solid by observing a very low amount of cerium leaching (<1.2 wt%) from the solid photocatalyst to the solution after three consecutive uses.

To put into context, the obtained values, the photocatalytic activity of Pt/Ce-UiO-66-NH_2_ for the OWS was compared with previous results. Caution should be taken when comparing the photocatalytic activity of several studies due to the relatively different reaction conditions employed, type of reactors and irradiation sources, presence or not of co-catalysts as well as use or not of homogeneous organic/inorganic redox relays among other possibilities. Regardless these comments, Table S5 in ESI† summarizes the use of MOF-based photocatalysts for the OWS into H_2_ and O_2_. From these results, it can be concluded that the Pt/Ce-UiO-66-NH_2_ photocatalyst is one of the most active MOF-based materials reported until now for the solar-driven photocatalytic OWS.^46^

## Conclusions

We report here a new scalable green room temperature synthesis approach, for the *de novo* versatile preparation of redox active Ce based MOFs, including the Ce-UiO-66-NH_2_ constructed from the redox active ligand 2-amino-1,4-benzenedicarboxylic acid. This was achieved through a kinetic control of the reducible character of the Ce(iv) species, while enabling the crystallization of the MOFs. Noteworthy, high quality Ce(iv)-MOF nanoparticles were formed with an excellent space time yield. The use of Ce-UiO-66-X solids as active photocatalysts for both HER and OER under simulated sunlight irradiation has then been investigated, finding that the relative photocatalytic HER and OER activity of the Ce-UiO-66-X series correlates well with the LUCO (HER) and HOCO (OER) band energy. Thus, the remarkable influence of terephthalate substituent on the LUCO/HOCO band energy values determines their relative photocatalytic activity with the Ce-UiO-66-NH_2_ and Ce-UiO-66-NO_2_ being the most active photocatalysts for HER and OER, respectively. Interestingly, these Ce-UiO-66-based materials were found to be more active than their zirconium analogues, due to the low-lying 4f orbitals of Ce^4+^ ions that favor the LMCT of the formers. These Ce-based materials could be reused at least during three consecutive times without observing loss of catalytic activity or crystallinity although some cerium leaching occurs. Based on the LUCO–HOCO band energy values as a function of terephthalate substituent, Ce-UiO-NH_2_ was the solid with the best aligned band energy values for OWS. The use of Pt NPs supported on the Ce-UiO-66-NH_2_ solid resulted in a highly active, stable and reusable photocatalyst for the OWS under simulated sunlight irradiation. These promising catalytic results may encourage future works dealing with fine tuning the energy-level diagram of Ce-UiO-66-X MOFs to accomplish the thermodynamic requirements of the photocatalytic water splitting process. Beyond the scope of Ce-MOFs, we expect the synthetic concept here shall guide the future rational and sustainable low temperature design of other highly challenging porous structures, such as Ti(iv) based MOFs.

## Data availability

All experimental data are provided in the ESI.†

## Author contributions

S. D., A. T., S. N., and C. S. conceived the concept of the project; S. D., E. M.-L., and H. G. B. performed the experiments. S. D., and S. N. wrote the original draft. S. D., S. N., H. G., A. T., and C. S. revised the manuscript. H. G. proposed the catalytic methodology. S. N., A. T. and C. S., led the project. All authors read and agreed on the content of the paper.

## Conflicts of interest

There are no conflicts to declare.

## Supplementary Material

SC-014-D2SC05161C-s001
