# Supplementary material for: Room temperature design of Ce(iv)-MOFs: from photocatalytic HER and OER to overall water splitting under simulated sunlight irradiation
Source: Chem Sci. 2023 Feb 24;14(13):3451–61. doi: 10.1039/d2sc05161c (PMC10055767; doi:10.1039/d2sc05161c)
Supplement: SC-014-D2SC05161C-s001 [file SC-014-D2SC05161C-s001.pdf]

Electronic Supplementary Material (ESI) for Chemical Science.
This journal is © The Royal Society of Chemistry 2023

# Supporting information

# Room Temperature design of Ce(IV)-MOFs: from photocatalytic HER and OER to overall water splitting under simulated sunlight irradiation

Shan Dai,[†] Eva Montero-Lanzuela,[§] Antoine Tissot,[†*] Herme G. Baldoví, [§] Hermenegildo García,† Sergio Navalón,[§*] Christian Serre[†*]

*† Institut des Matériaux Poreux de Paris, UMR 8004 Ecole Normale Supérieure, ESPCI Paris, CNRS, PSL University, 75005, Paris, France*

*[§] Departamento de Química, Universitat Politècnica de València, C/Camino de Vera, s/n, 46022, Valencia, Spain*

*† Instituo de Tecnología Química (CSIC-ITQ), Av de Los Naranjos, s/n, 46022, Valencia, Spain*

**Chemicals.**

All chemicals were purchased from commercial suppliers and used as received without further purification. Terephthalic acid, 99%, Alfa Aesar. 2-Aminoterephthalic acid (BDC-$NH_2$), 99+%, Acros. 1,2,4-Benzenetricarboxylic acid (BDC-COOH), 98%, Acros. 1H-Pyrazole-3,5-dicarboxylic acid(PDA), 97%, FluoroChem. 2-Bromoterephthalic acid, 98%, Acros, Ammonium Cerium(IV) nitrate, 99%, Acros. Acetic acid, 99%, Acros. Formic acid, 98+%, Acros. Ethanol absolute, >=99%, Acros. MilliQ water, Millipore system.

## Instruments

Powder X-ray Diffraction (PXRD) data were recorded on a high-throughput Bruker D8 Advance diffractometer working on transmission mode and equipped with a focusing Göbel mirror producing CuKα radiation (λ = 1.5418 Å) and a LynxEye detector. Nitrogen porosimetry data were collected on a Micromeritics Tristar/ Triflex instrument at 77K (pre-activating samples at 80°C under vacuum, 12 hours). SEM images were recorded either with FEI Magellan 400 scanning electron microscope. TGA data were collected on Mettler Toledo TGA/DSC 2, STAR System apparatus with a heating rate of 5 °C/min under the oxygen flow. Infrared spectra were measured with a Nicolet iS5 FTIR ThermoFisher spectrometer. The optical spectra of solids have been measured using a Varian Cary 300 Bio UV-Vis spectrometer equipped with an integration sphere in diffuse reflectance mode. X-ray photoelectron spectra (XPS) of the solids were recorded using a SPECS spectrometer equipped with an MCD-9 detector using a monochromatic Al (Kα = 1486.6 eV) X-ray source. CASA software has been employed for the high-resolution XPS deconvolution.

## Synthesis of MOFs.

**Room temperature strategy (RTS) for Ce-UiO-66-X:** 1.5 mmol (820 mg) of $(NH_4)_2Ce(NO_3)_6$ was weighted in a glass vial, 3 mL of acetic acid and 8 mL of distilled water were stepwisely introduced in the reactor, following by 1 minute of stirring at 600 rpm. 1.5 mmol of corresponding ligand (2-Bromoterephthalic acid/ 2-Nitroterephthalic acid/ 1,2,4-Benzenetricarboxylic acid) was subsequently added in the solution. The solution became very cloudy within 1-2h of stirring at room temperature, indicating the formation of the corresponding MOF. The product was collected by centrifugation, washed with water and ethanol and finally dried under vacuum. The product yield was 89% for Ce-UiO-66-Br, 82%

for Ce-UiO-66-$NO_2$, 80% for Ce-UiO-66-COOH based on the Ce(IV) salt due to the excess of ligand.

**Synthesis of Ce-UiO-66 and Ce-UiO-66-$NH_2$:** the synthesis of these two MOFs followed the RTS while adding 20 mL Ethanol in the solution before the introduction of the corresponding ligands (Terephthalic acid/ 2-aminoterephthalic acid). The product yield was 92% for Ce-UiO-66 based on the Ce(IV) salt due to the excess of ligand, 42% for Ce-UiO-66-$NH_2$ based on the ligand due to excess of Ce(IV) salt.

**Synthesis of Ce-DUT-67 (PDA): t**he synthesis of Ce-DUT-67(PDA) followed the RTS while using 1 mmol of 1H-Pyrazole-3,5-dicarboxylic acid monohydrate (PDA) instead of 1.5 mmol ligands used for RTS. The product yield was 65% based on the Ce(IV) salt.

**Synthesis of Ce-MOF-808: t**he synthesis of Ce-MOF-808 followed the RTS while using 0.5 mmol of Benzene-1,3,5-tricarboxylic acid (BTC) and 3 mL formic acid instead of 1.5 mmol ligands used for RTS. The product yield was 86% based on the Ce(IV) salt.

**Preparation of Pt NPs on UiO-66(Ce)-$NH_2$.**

Pt NPs were deposited in the previously formed Ce-UiO-66-$NH_2$ solid using the photodeposition method. [1] Ce-UiO-66-$NH_2$ (50 mg) was dispersed in a mixture of Milli-Q water (13 mL) and methanol (5 mL) using a quartz tube. Then, the corresponding amount of hexachloroplatinic (IV) acid hexahydrate previously dissolved in water (1 mL) was added to this quartz tube and the system purged with argon for 30 min. The system was irradiated using a UV-vis light lamp (150 W) for 3 h. The resulting solid was filtered, washed several times with Milli-Q water and dried in an oven at 100 ºC for 24 h.

**Photocatalysis details.**

Photocatalytic hydrogen evolution reaction. Briefly, a certain amount of MOF as photocatalyst (20 mg) was placed in a quartz reactor (51 mL) containing a mixture of methanol (4 mL) as electron donor and Milli-Q water (16 mL). The system was sonicated (450 W) for 15 min to achieve a good dispersion. Subsequently, this suspension was purged with argon for 60 min to remove air from the reactor. The MOF suspension under stirring was irradiated under simulated sunlight irradiation (150 W Hamamatsu Hg-Xe lamp ref L8253; Hamamatsu spot light source L9566-04; Hamamatsu light guide A10014-50-0110; Lasing AM 1.5G type filter ref. 81094). The evolved gases were analyzed at the required period of time from the head space of the quart reactor by direct connection of the reactor to a Micro GC system (Agilent 490 Micro GC system equipped with a Molsieve 5 Å column) that employ argon as carrier gas. During the photocatalytic experiments the temperature of the system was monitored, and the pressure measured by using a manometer adapted to the reactor.

Photocatalytic oxygen evolution reaction. These experiments were performed as described for the HER but using as reaction mixture a Milli-Q water (20 mL) solution of potassium persulfate (700 mg) as electron acceptor.

Photocatalytic overall water splitting reaction. These experiments were performed as described for the HER but using Milli-Q water (20 mL)

The photocatalytic experiments for the HER, OER and OWS were carried out at least in triplicate trials. The presented photocatalytic data for these reactions is the average of the three independent experiments.

**Photophysical measurements**

Initially, a stock dispersion of UiO-66(Ce)-$NH_2$ or Pt/UiO-66(Ce)-$NH_2$ in acetonitrile (1 mg/10 mL) was prepared and the dispersion was sonicated for 15 min (450 W). Then, a volume of each MOF dispersion (ca 0.2 mL) was further diluted with acetonitrile (ca 2.8 mL).

The resulting suspensions were analyzed by UV-Vis spectroscopy and diluted with acetonitrile to reach an absorbance at 360 nm of 0.35 optical density. These MOFs suspensions were purged in argon for 15 min and immediately analyzed by photoluminescence (PL) or laser flash photolysis spectroscopies using 10 × 10 mm quartz cells. PL spectroscopy measurements were carried out with JASCO FP-8500 instrument. Steady-state fluorescence or PL measurements were performed with a FLS1000 spectrofluorometer (Edinburgh Instruments, Livingston, UK), equipped with an N-DMM double-emission monochromator, an N-G11 PMT-980 detector and a pulsed Xe lamp (450W) as the excitation source. Laser flash photolysis experiments were performed using an OPO System Ekspla (EKS-NT342C-10) coupled with an UV extension (EKS-NT342C-SH-SFG) as the excitation source and an Edinburgh Instruments detection System (LP980) coupled with an ICCD camera (Andor iStar CCD 320T). In this case, OPO System Ekspla light sources was adjusted to 268 nm. For this experiment, we prepared suspensions with 0,4 absorbance at 268 nm. When quenchers were utilized, suspensions were purged with pure $O_2$ or $N_2O$ for 10 min. In the case of methanol, 100 μL were introduced into the cuvette with a needle and the purged with argon during 10 min.

**Band energy values determination**

The optical band gaps of the series of Ce-UiO-66-X and Ce-MOF-808were estimated from the UV-Vis diffuse reflectance spectroscopy data using the Tauc plot. Specifically, the estimated direct and indirect band gaps were determined by using the following equation:

$$\alpha h\nu = k\left(h\nu - E_g\right)^{n/2}$$

For the estimation of the band gap the Y axis corresponds to the $(\alpha h\nu)^2$ or $(\alpha h\nu)^{1/2}$ values for the direct or indirect bandgap, respectively, and the X axis is hν. In this equation α is the absorption coefficient, h is the Planck constant, ν is the light frequency, k is a constant, and Eg is the band gap.

The valence band maxima of of Ce-UiO-66-X and Ce-MOF-808 versus the Fermi level ($E^f_\upsilon$) were determined by XPS. Then, the valence band position with respect to the NHE ($E_\upsilon^{NHE}$) was obtained from the following equation $E_\upsilon^{NHE} = E_\upsilon^{f} + \phi_{sp} + E_0^{SHE}$, where $\phi_{sp}$ is the work function of the spectrometer used for the measurements (4.244 eV) and $E_0^{SHE}$ is the energy of the SHE with respect to vacuum level of the electron with the value of −4.44 eV. From the corresponding $E_\upsilon^{NHE}$ value and the optical band gap, the conduction band energy minimum ($E_c^{NHE}$) of each material can be determined.

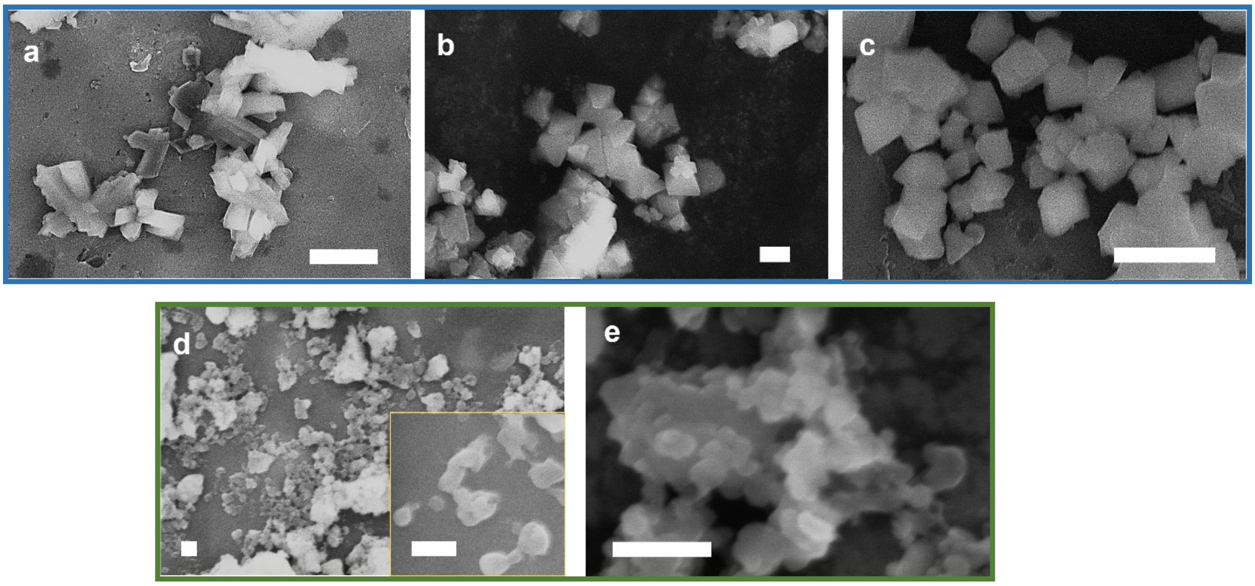


**Figure S1.** SEM images of a) Ce-UiO-66, b) Ce-UiO-66-Br, c) Ce-UiO-66-COOH, scale bar a-c= 1 μm; d) Ce-UiO-66-$NH_2$, e) Ce-UiO-66-$NO_2$, scale bar d, e= 200 nm.

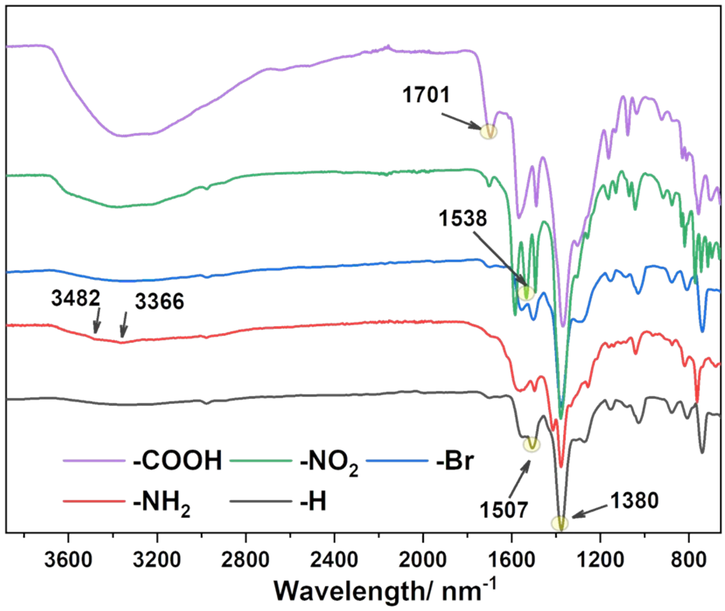


**Figure S2.** FTIR spectra of Ce-UiO-66-X (X=H, $NH_2$, Br, $NO_2$, COOH).

**Table S1.** Assignment of characteristic vibration bands in the FTIR spectra of the prepared Ce-UiO-66-X (X=H, $NH_2$, Br, $NO_2$, COOH).

| **IR vibration bands ($cm^{-1}$)** | **Assignment** |
|---|---|
| 3482, 3366 | N-H stretches of primary amines |
| 1701 | $\nu_{sym}$ (C=O), uncoordinated COOH |
| 1538 | $\nu_{asy}$ (N-O), nitro group |
| 1507, 1380 | Vibration of terephthalate ion, acetate ion |

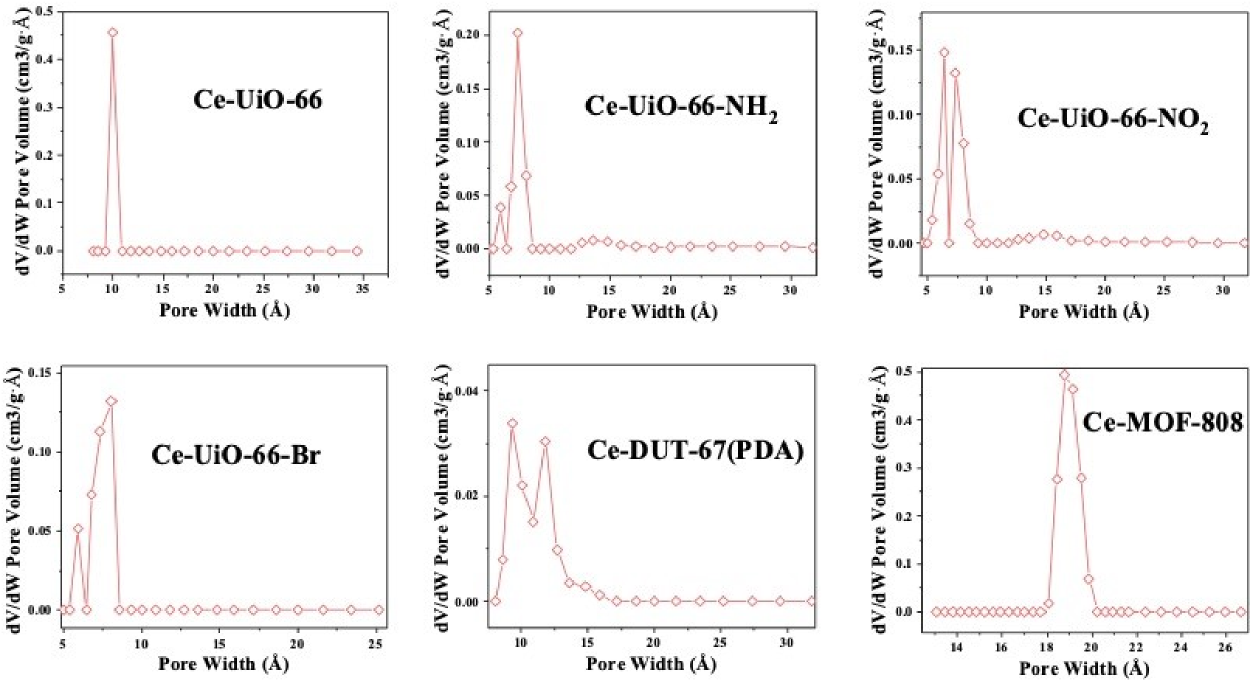


**Figure S3.** Pore size distribution of the MOFs in this work obtained from 77K N2 isotherms using a DFT model.

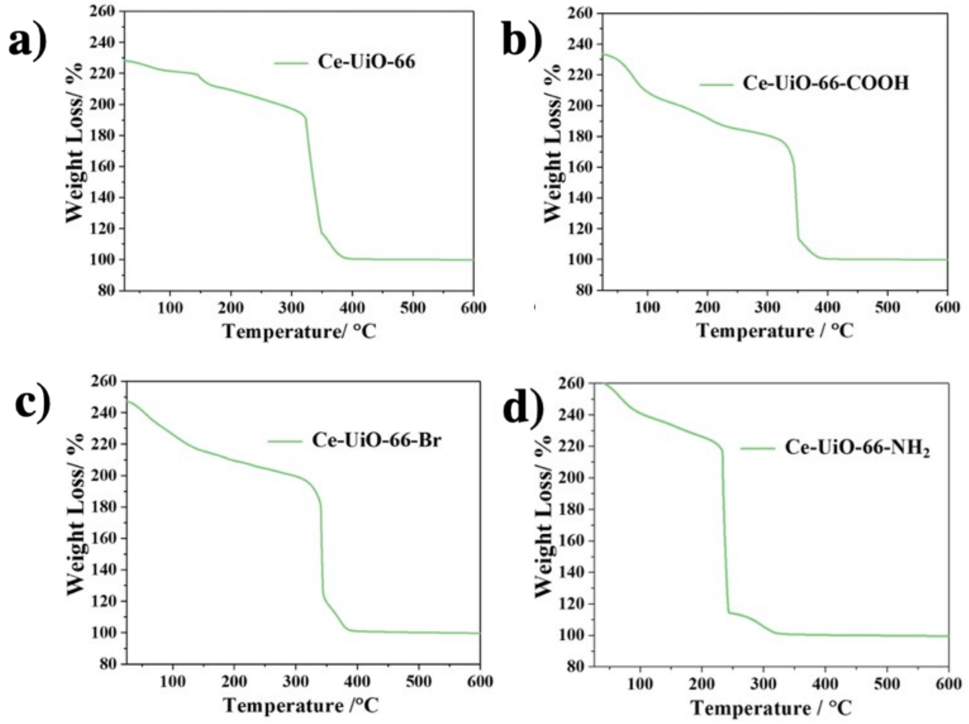


**Figure S4.** TG analysis of Ce-UiO-66-X (X= $NH_2$, H, Br, COOH). Normalized with the residual mass at 600 °C ($CeO_2$).

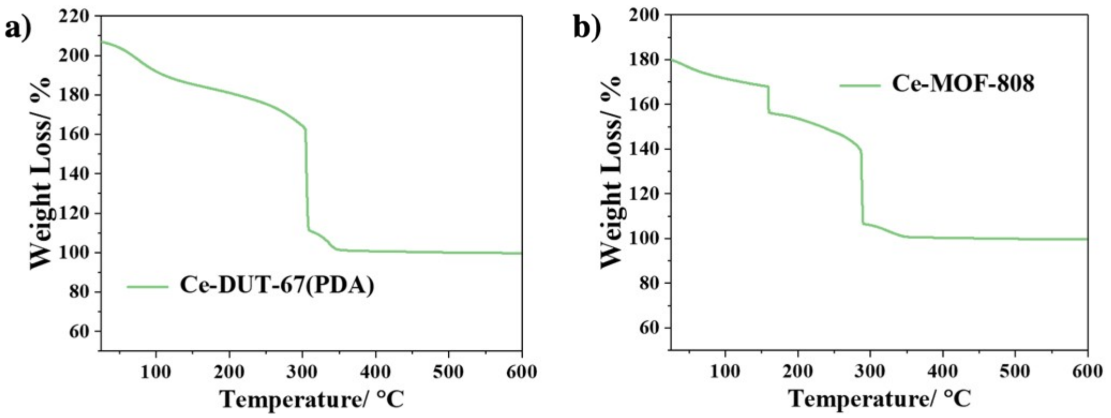


**Figure S5.** TG analysis of Ce-DUT-67(PDA) and Ce-MOF-808. Normalized with the residual mass at 600 °C ($CeO_2$).

**Table S2.** Comparison of the theoretical mass loss (from structure) and measured mass loss of Ce-MOFs in this work. (extracted from TGA)

* The mass loss here has been normalized with the residual $CeO_2$ for easier comparison.

** The theoretical mass loss was calculated according to the intact structure of Ce-UiO-66-X ($Ce_6O_4(OH)_4(Linker)_6$). The fully thermally decomposed residue is considered as $CeO_2$ and the mass of residue is normalized to 100%. Subsequently, the theoretical mass content of organic linker is calculated on the basis of 100%.

| Entry | Materials | T/ $^{\circ}C$ | Measured* wt% | Theoretical** wt% |
|---|---|---|---|---|
| 1 | Ce-UiO-66 | 325 | 192 | 195 |
| 2 | Ce-UiO-66-$NH_2$ | 225 | 220 | 204 |

| | | | | |
|---|---|---|---|---|
| 3 | Ce-UiO-66-$NO_2$ | 295 | -- | 213 |
| 4 | Ce-UiO-66-Br | 325 | 198 | 237 |
| 5 | Ce-UiO-66-COOH | 320 | 180 | 220 |
| 6 | Ce-DUT-67(PDA) | 300 | 170 | 160 |
| 7 | Ce-MOF-808 | 280 | 140 | 140 |

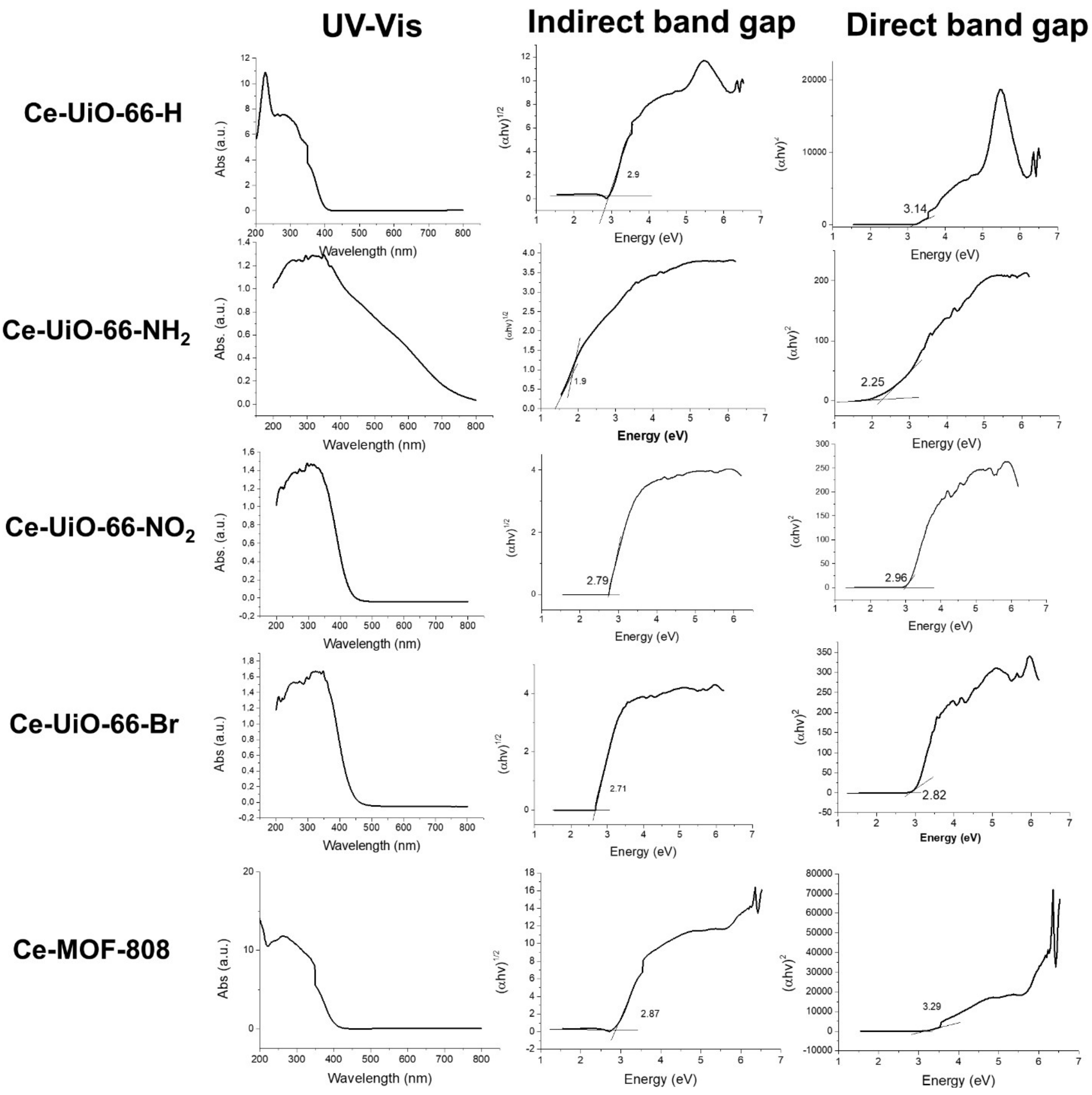


**Figure S6.** UV-Vis reflectance spectra and the derived Tauc plots (indirect and direct bandgap) of Ce-UiO-66-X (X=H, $NH_2$, Br, $NO_2$, COOH) and Ce-MOF-808.

**Table S3.** Summary of the direct and indirect band gap values estimated in this work and comparison with reported studies.

| | Direct band gap (Ev) | Indirect band gap (eV) | Other reports |
|---|---|---|---|
| Ce-UiO-66-H | 3.14 | 2.9 | 2.82 eV direct. [2] |
| Ce-UiO-66-$NH_2$ | 2.25 | 1.9 | 2.09 eV direct. [2] |
| Ce-UiO-66-$NO_2$ | 2.96 | 2.79 | 2.79 eV direct. [2] |
| Ce-UiO-66-Br | 2.82 | 2.71 | 2.68 eV direct. [2] |
| Ce-MOF-808 | 3.12 | 2.87 | - |

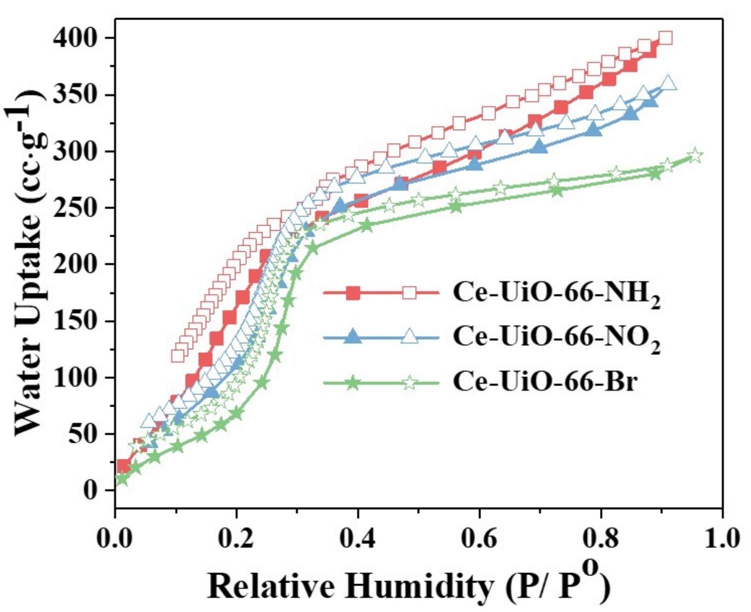


**Figure S7.** Water sorption isotherms of Ce-UiO-66-$NH_2$/ $NO_2$/ Br at 25°C.

## Ce-UiO-66-H

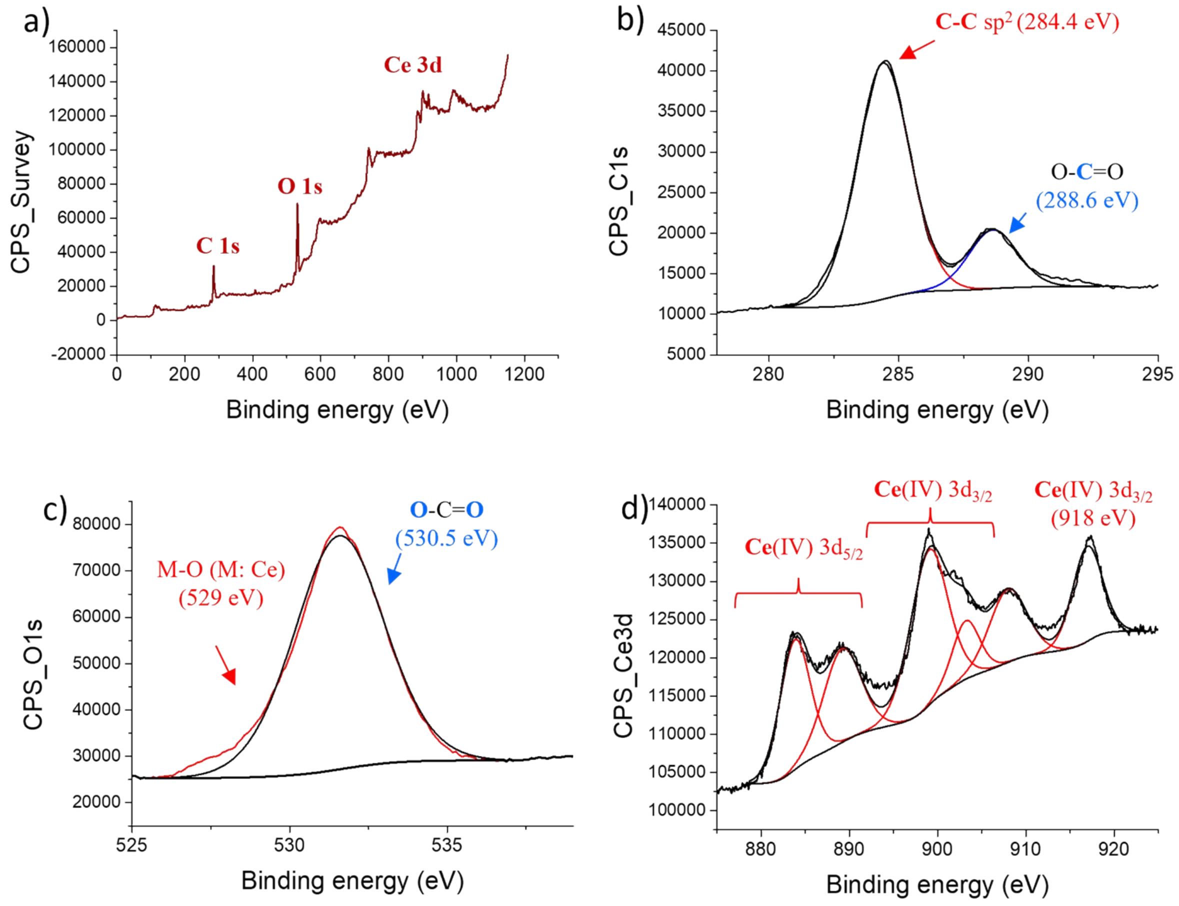


**Figure S8.** Survey and high-resolution XPS spectra of Ce-UiO-66-H.

## $Ce-UiO-66-NH_2$

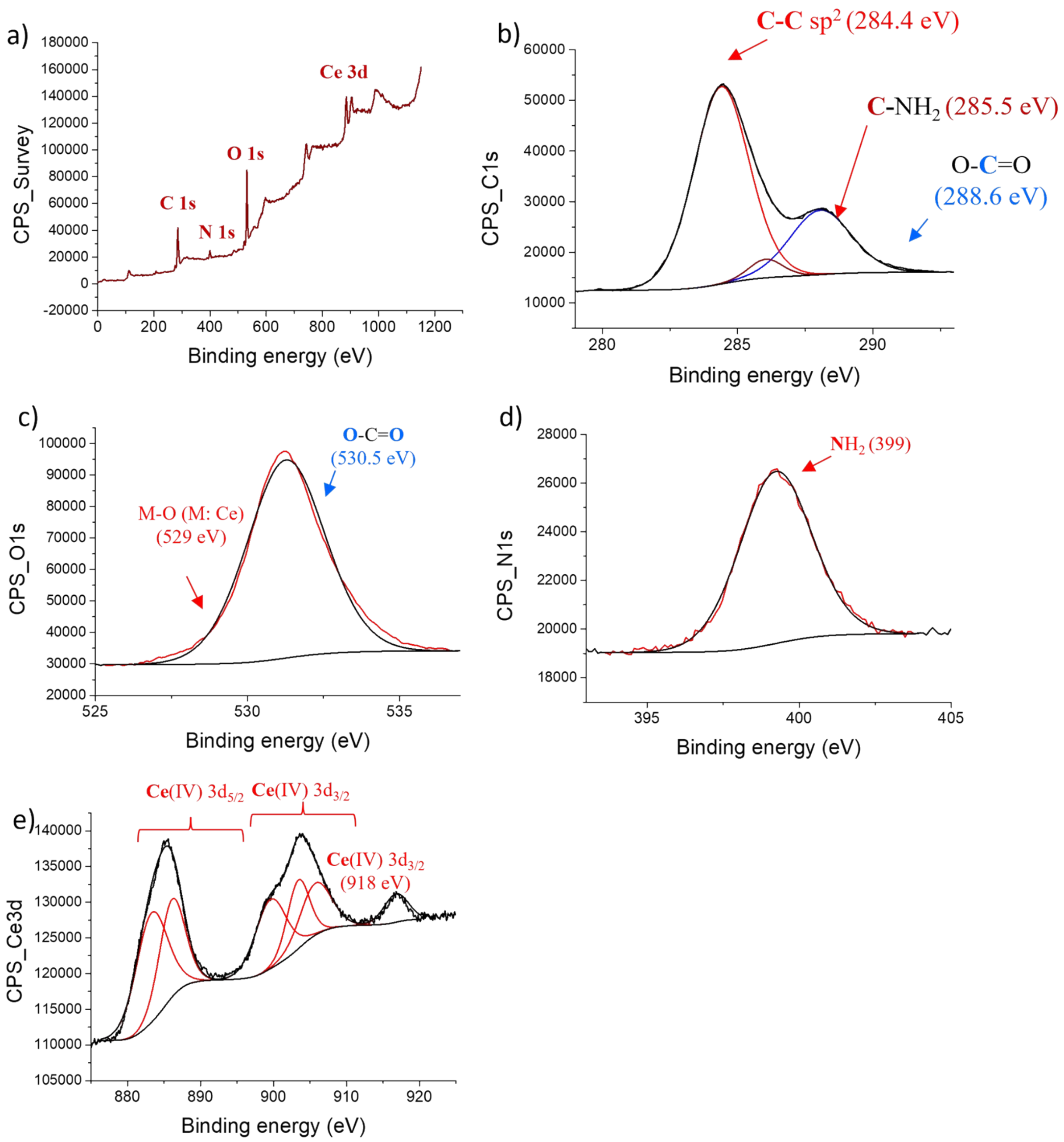


**Figure S9.** Survey and high-resolution XPS spectra of $Ce-UiO-66-NH_2$.

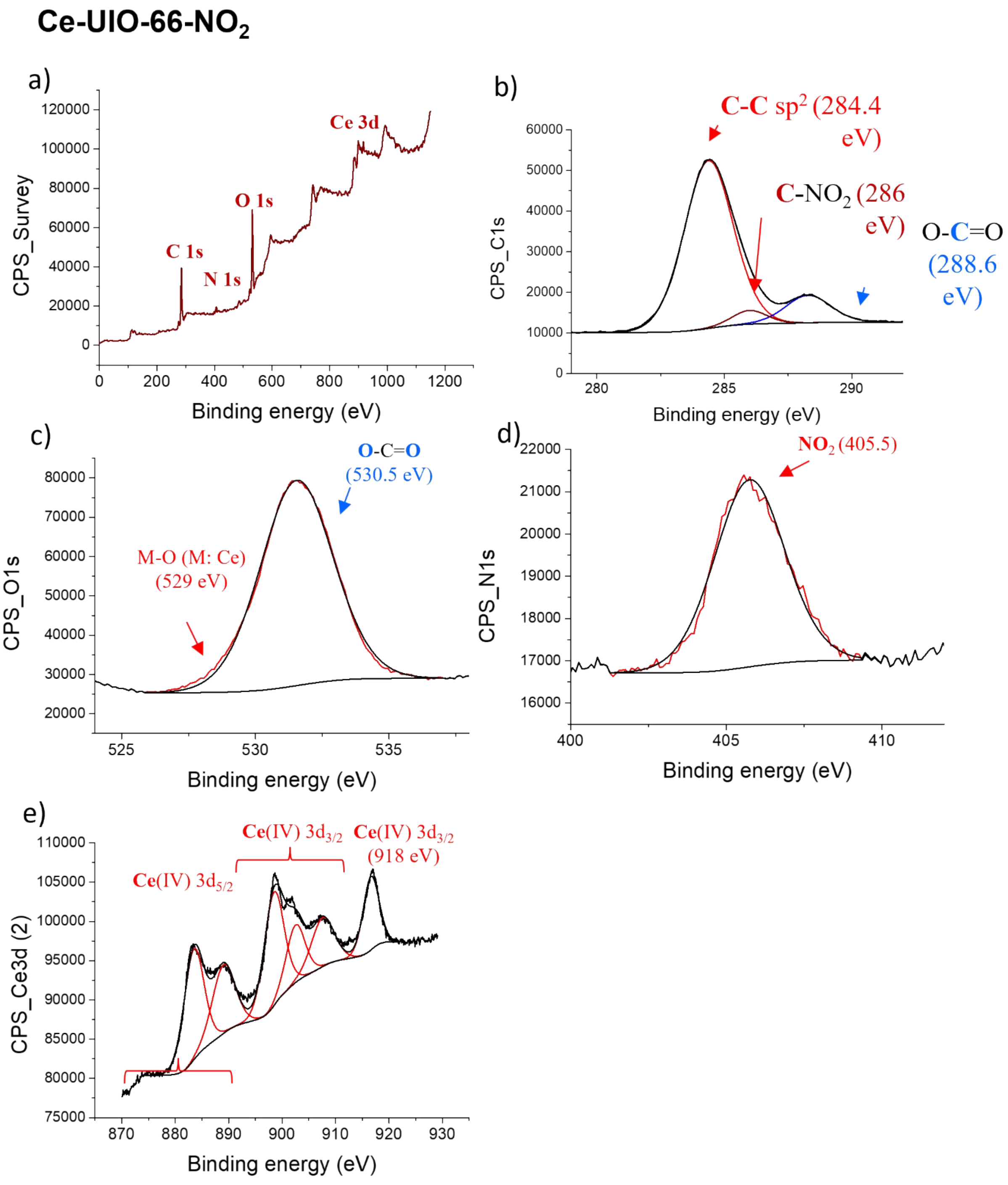


**Figure S10.** Survey and high-resolution XPS spectra of Ce-UiO-66-$NO_2$.

**Ce-UiO-66-Br**

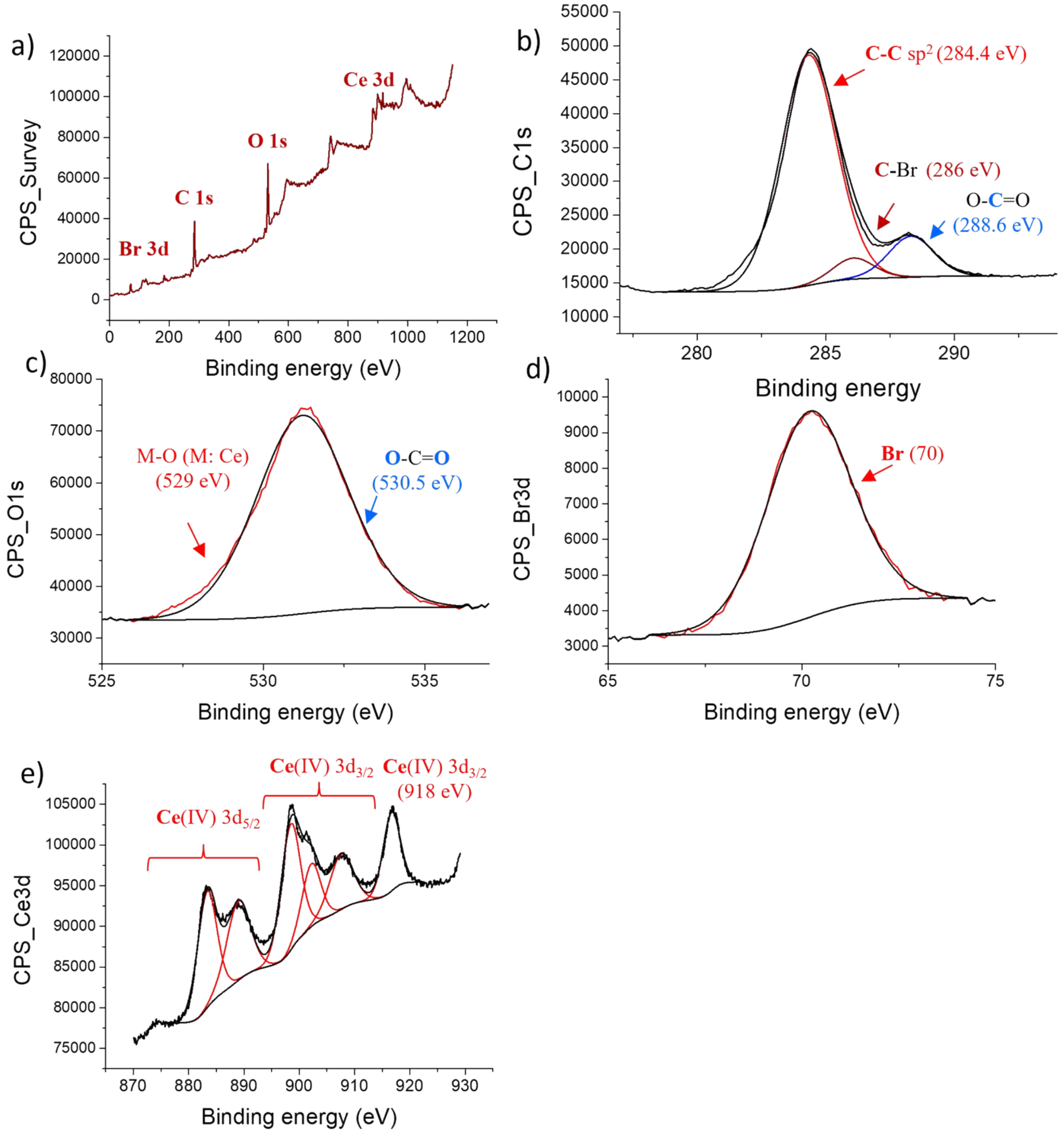


**Figure S11.** Survey and high-resolution XPS spectra of Ce-UiO-66-Br.

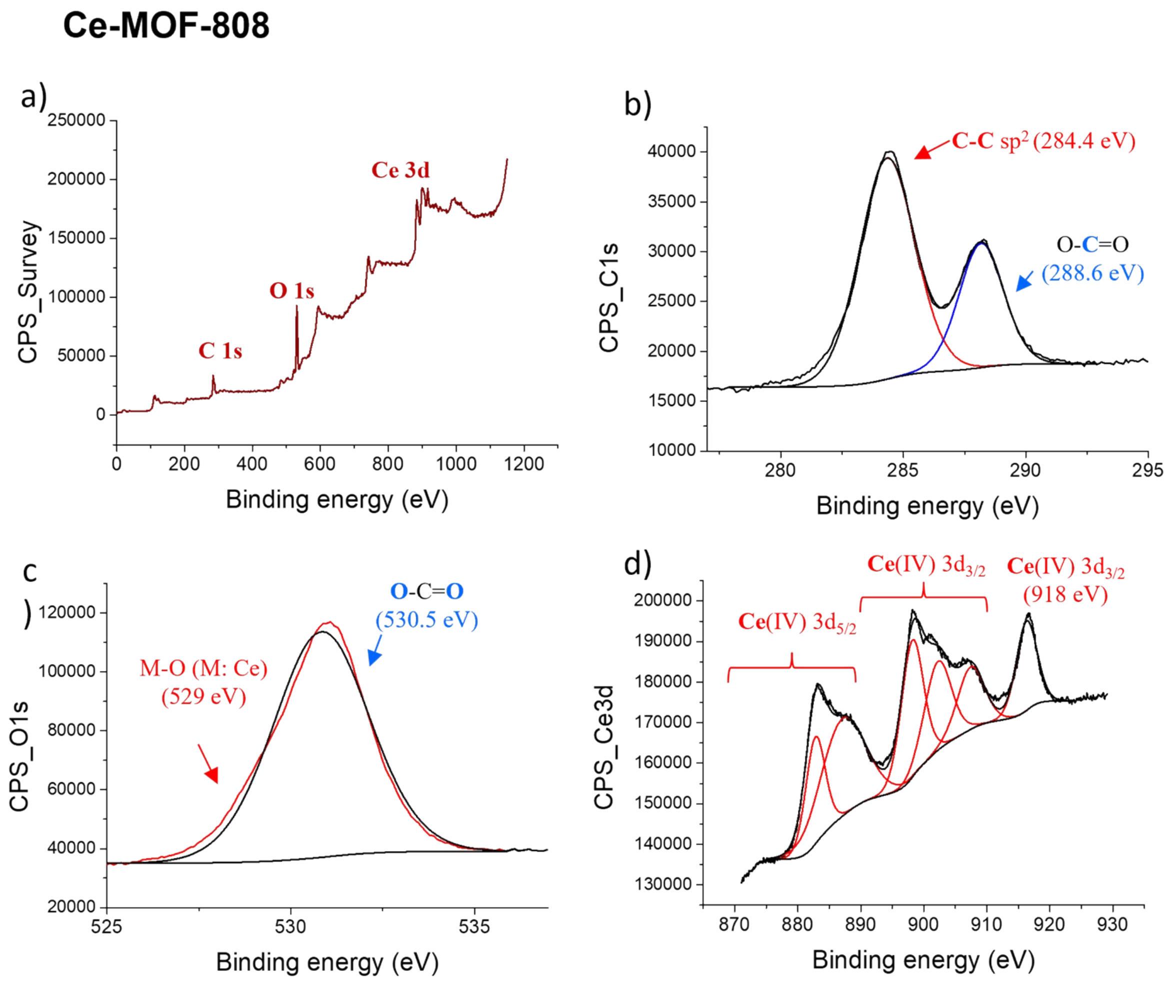


**Figure S12.** Survey and high-resolution XPS spectra of Ce-MOF-808.

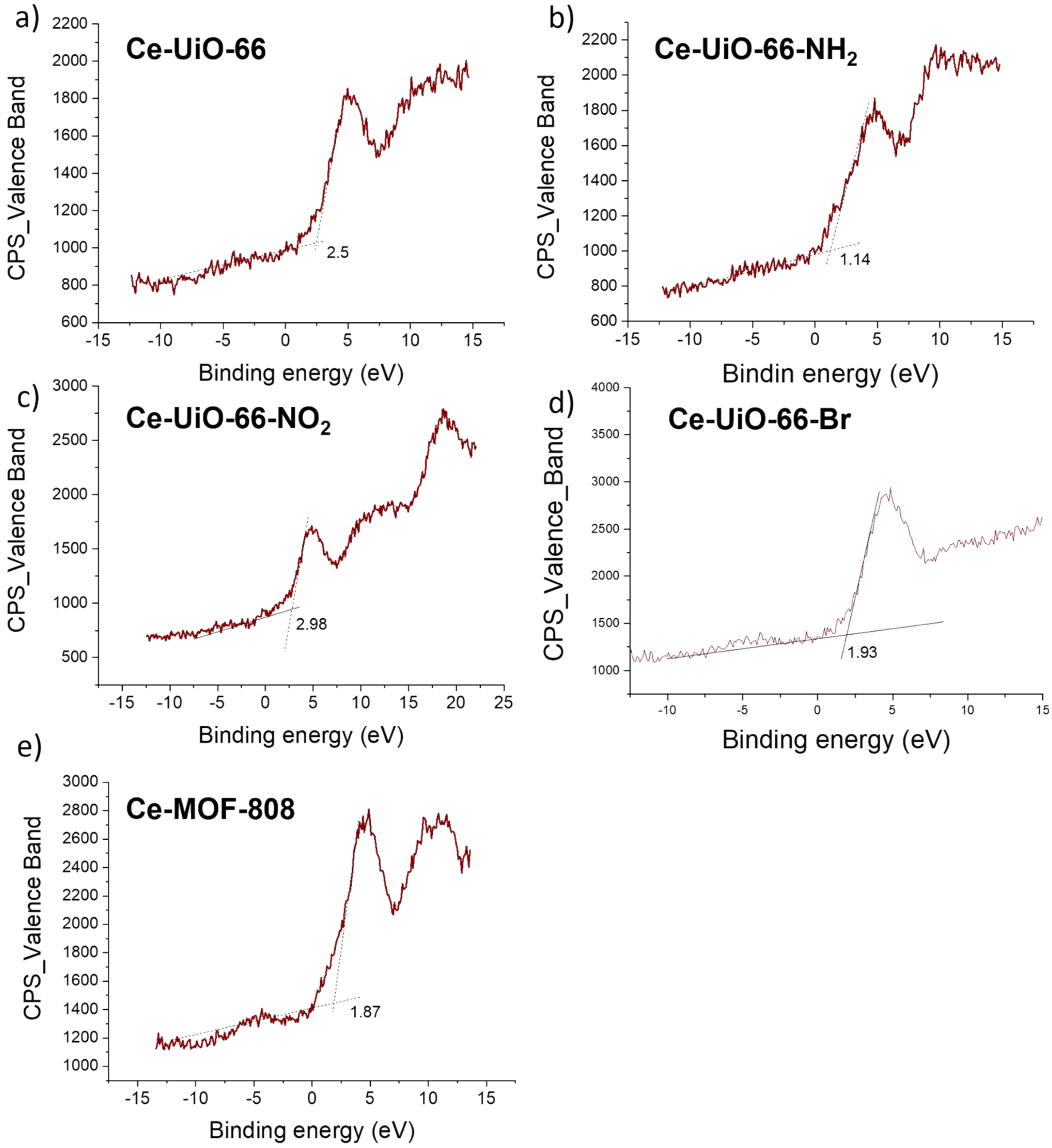


**Figure S13.** XPS valence band spectra of the synthesized Ce-MOFs in this work. The valence band is achieved by extrapolating a linear fit to the leading edge of the spectra to the baseline.

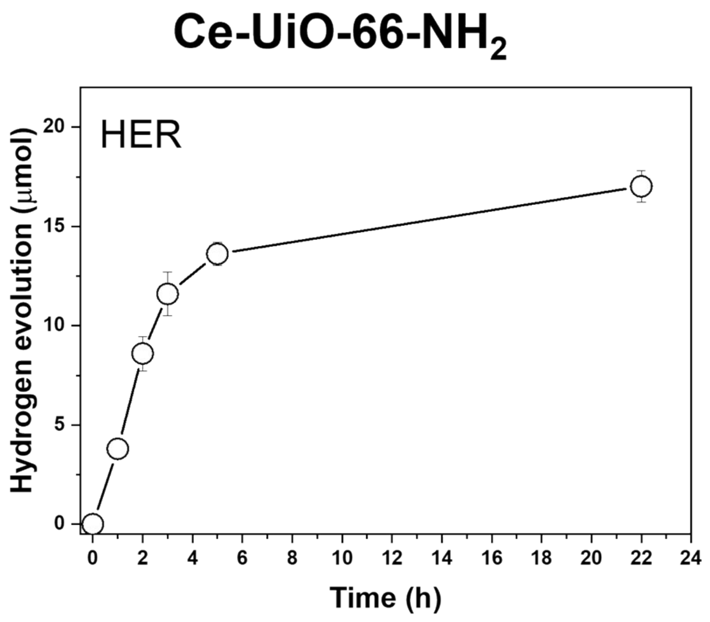


**Figure S14.** Photocatalytic HER using Ce-UiO-66-$NH_2$.

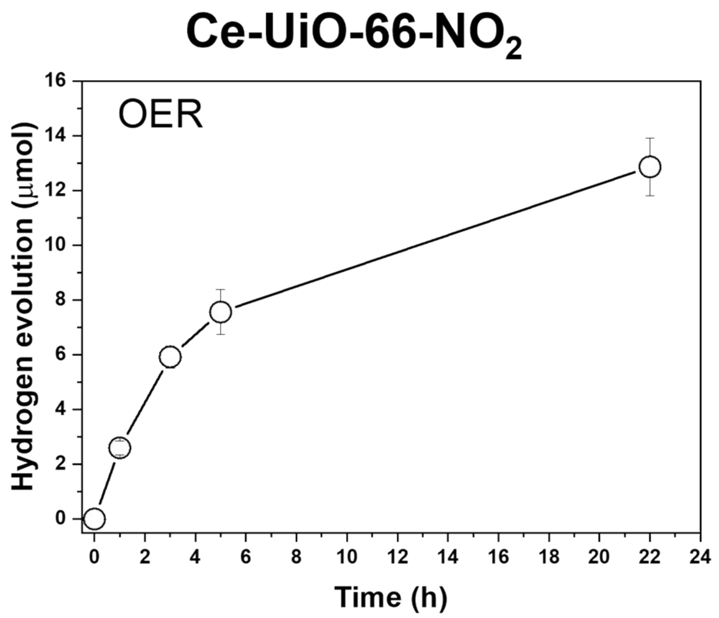


**Figure S15.** Photocatalytic OER using Ce-UiO-66-$NO_2$.

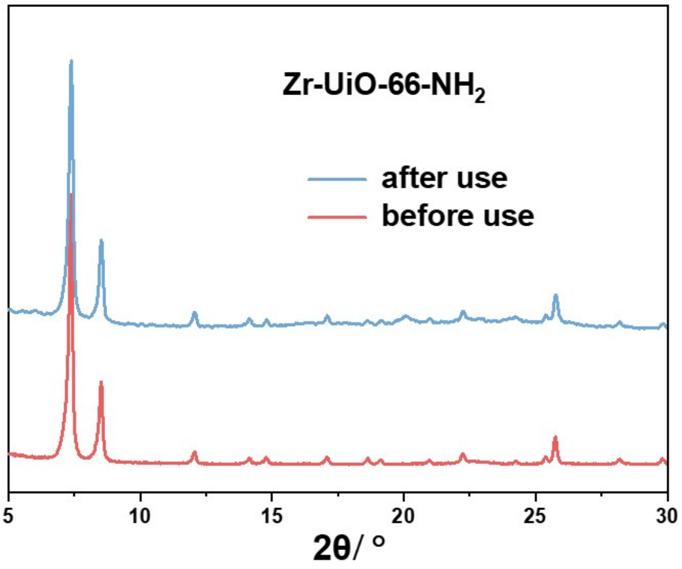


**Figure S16.** PXRD pattern ($\lambda_{Cu}$=1.5406Å) of the Zr-UiO-66-NH$_2$ after and before catalysis.

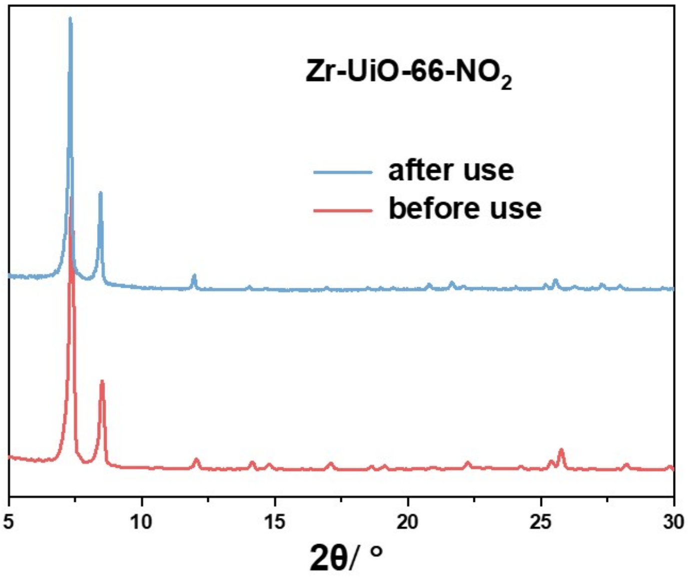


**Figure S17.** PXRD pattern ($\lambda_{Cu}$=1.5406Å) of the Zr-UiO-66-NO$_2$ after and before catalysis.

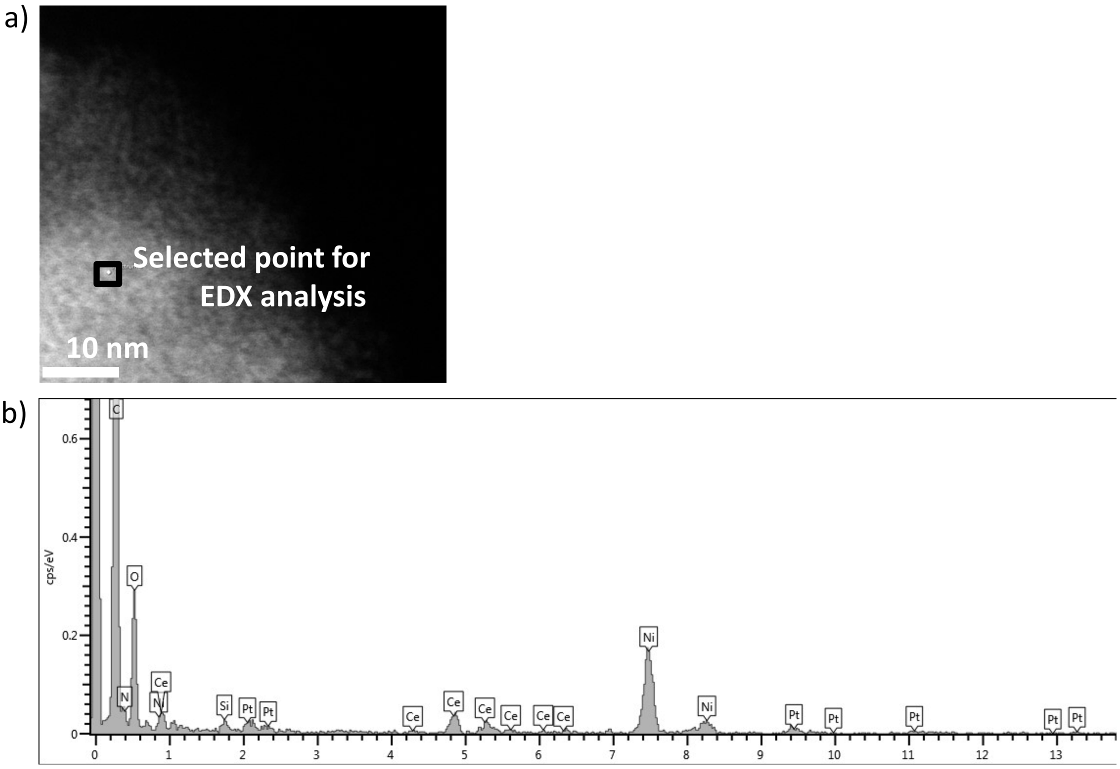


**Figure 18.** Representative DF-STEM image of Pt/Ce-UiO-66-$NH_2$ (a) and EDX spectrum form selected point in panel a (b).

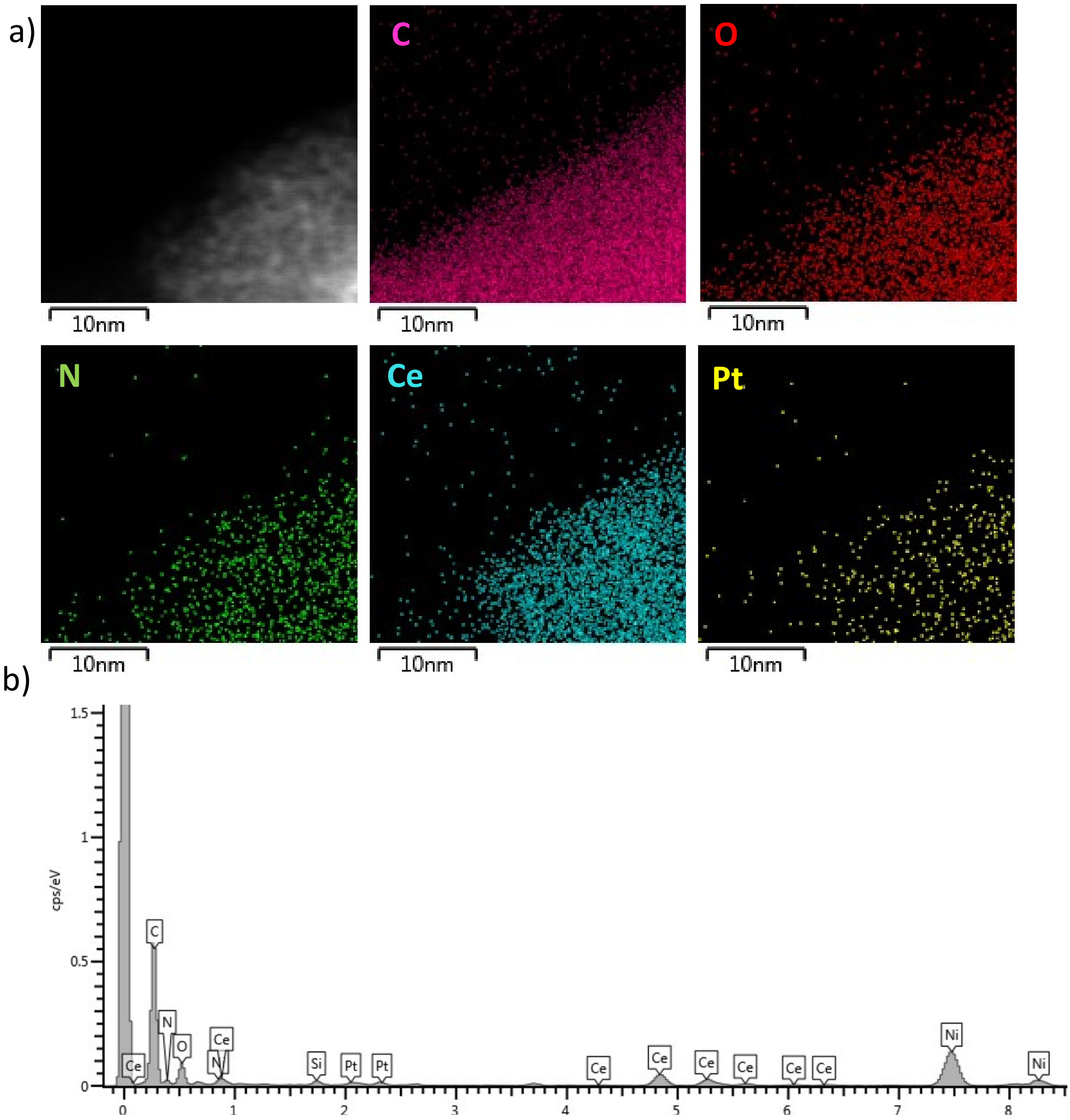


**Figure S19.** DF-STEM image (a), element mapping (C, O, N, Ce and Pt) (b) and EDX spectrum of a representative are of Pt/Ce-UiO-66-$NH_2$. Note: The presence of nickel in the EDX spectrum due to the nickel grid employed for the analysis.

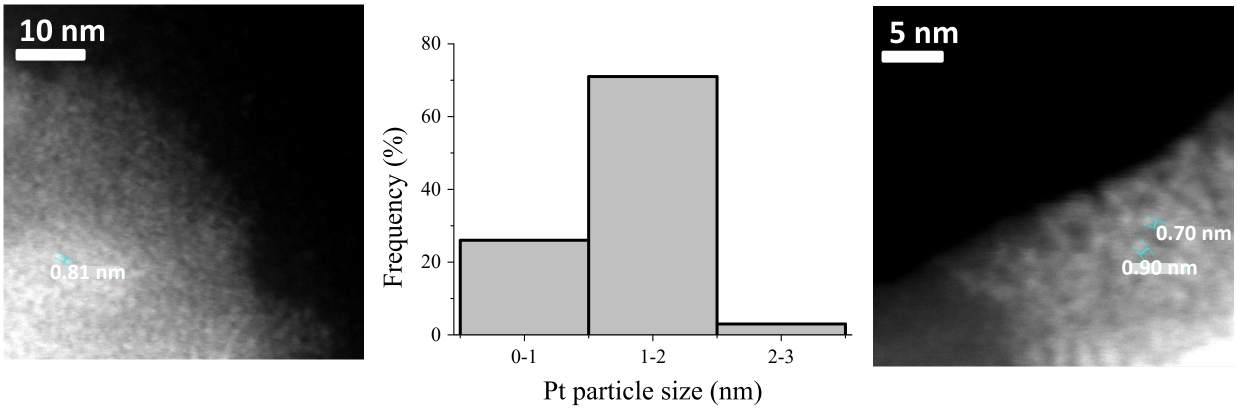


**Figure S20.** Representative DF-STEM images and platinum particle size distribution of Pt/Ce-UiO-66-$NH_2$.

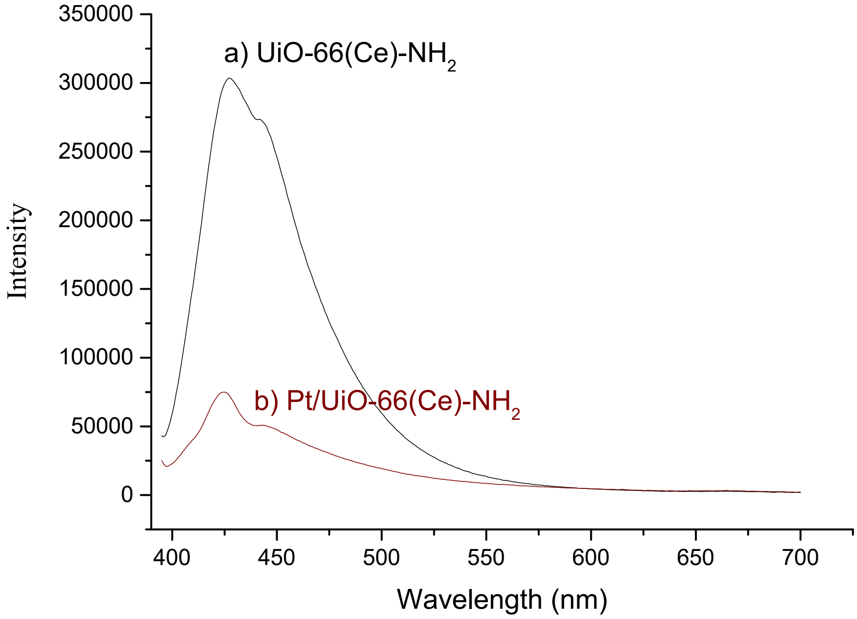


**Figure S21.** PL emission spectra ($\lambda ex$ = 340 nm ) of acetonitrile suspensions of Ce-UiO-66-$NH_2$ and Pt/Ce-UiO-66-$NH_2$.

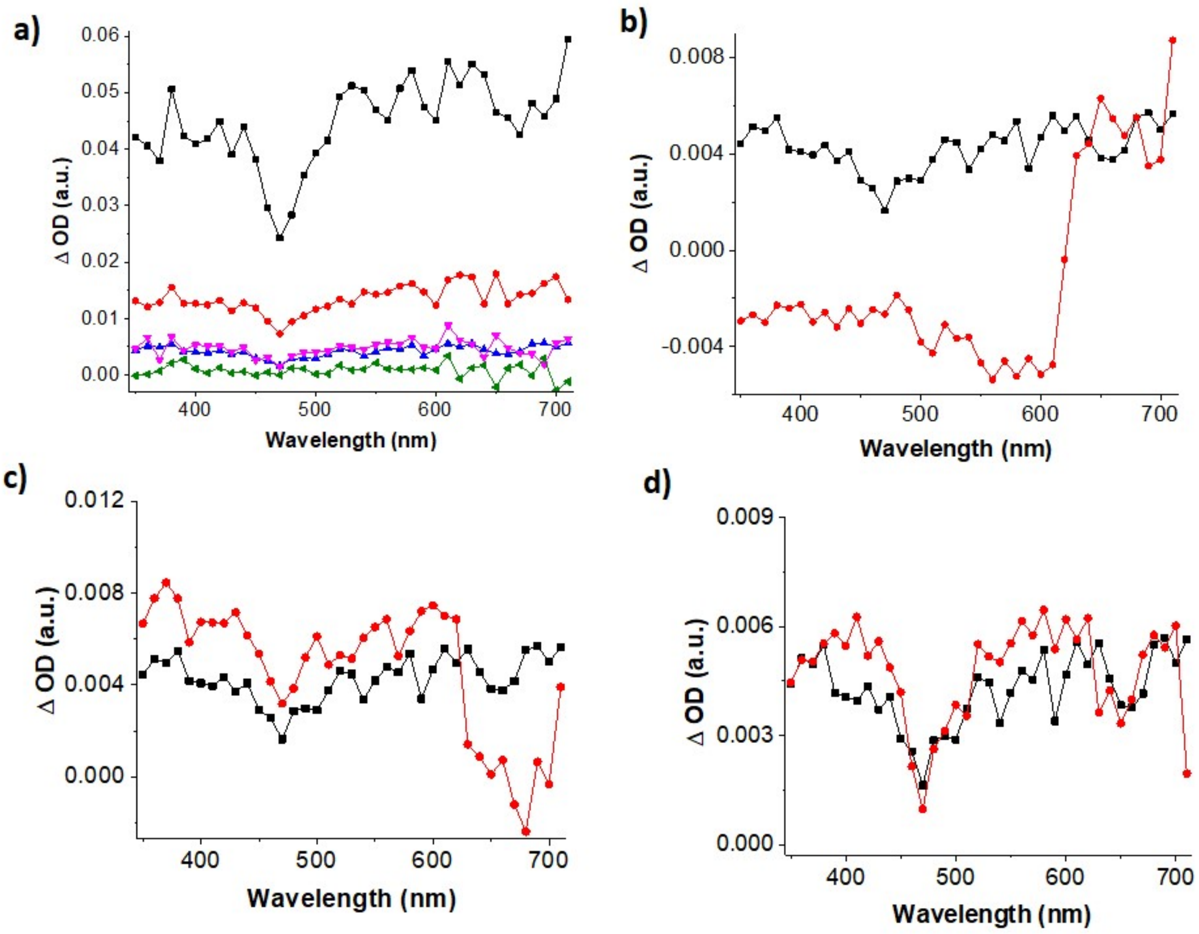


**Figure S22.** a) Transient absorption spectra of Pt/Ce-UiO-66-$NH_2$ under Ar atmosphere at different times (■ 37 ns, ● 144 ns, ▲ 500 ns, ▼ 1600 ns and◄ 4100 ns). Comparison of transient absorption spectra of argon (■) at 500 ns with different quenchers MeOH (b, circle red), $O_2$ (c, circle red) or $N_2O$ (d, circle red).

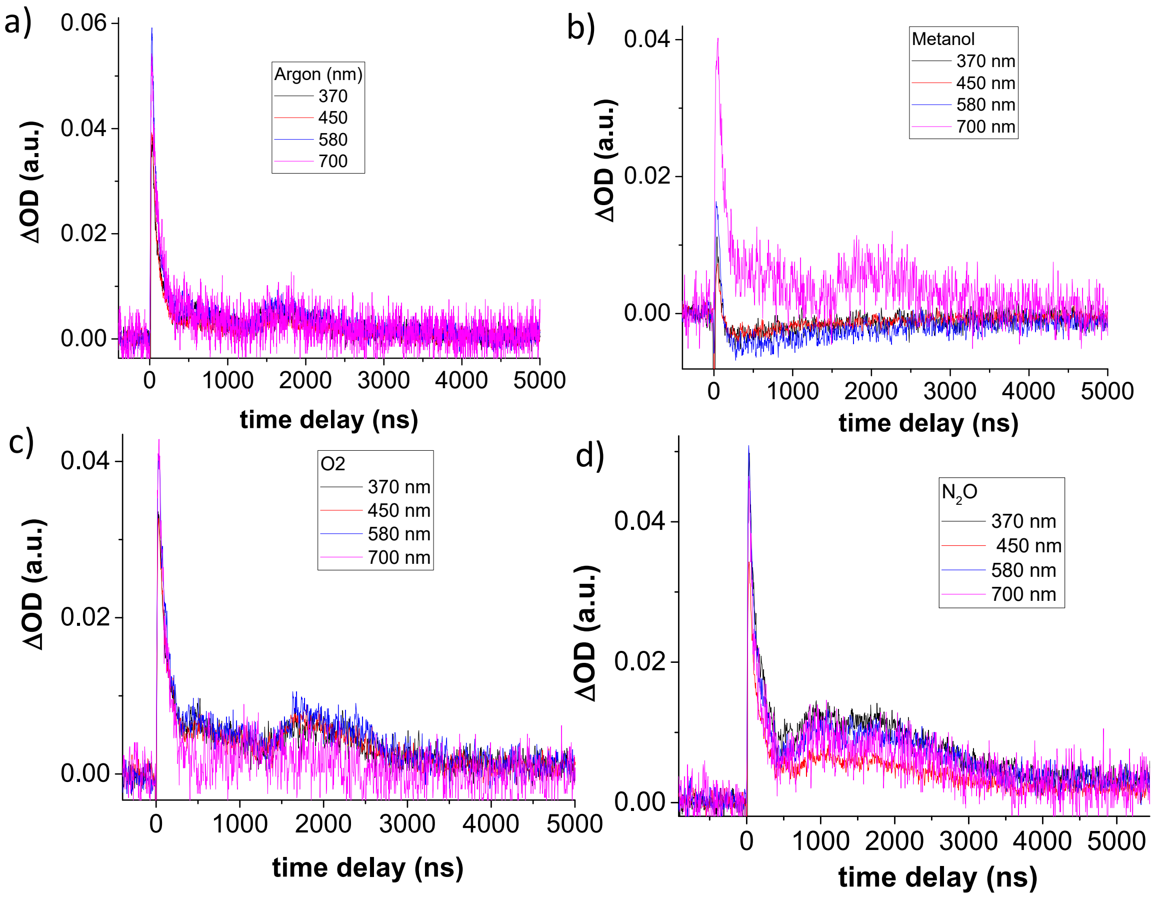


**Figure S23.** Decay profiles of Pt/Ce-UiO-66-$NH_2$ from TAS spectra from Figure S18 measured under (Ar), MeOH (b), $O_2$ (c) and $N_2O$ (d) at different wavelengths (370, 450, 580 and 700 nm).

**Table S4** $H_2/O_2$ molar ratio measured during the photocatalytic overall water splitting in three consecutive cycles using Pt@Ce-UiO-66-NH2 as photocatalyst (data corresponding to the temporal gas evolution during photocatalytic OWS, Figure 7b in the main text)

| | 1st use | 2nd use | 3rd use |
|---|---|---|---|
| 0 | 0 | 0 | 0 |

| | | | |
|---|---|---|---|
| 2 | 5.6 | 6.3 | 6.5 |
| 5 | 2.7 | 4.1 | 3.4 |
| 22 | 2.6 | 2.6 | 2.9 |

**Table S5** Photocatalytic activity for the OWS using MOF-based under natural or simulated sunlight irradiation or visible light irradiation.

| Catalyst | Co-catalyst | Reaction conditions | Photocatalytic activity | Reference |
|---|---|---|---|---|
| Ce-UiO-66-$NH_2$. | Pt NPs | Photocatalyst (20 mg), $H_2O$ (20 mL), simulated sunlight irradiation (Xe-Hg lamp 150 W, 1.5 AM filter), 35 ºC, 22 h | 205 and 80 µmol $g^{-1}$ for $H_2$ and $O_2$, respectively | This work |
| Ti-MOF (IEF-11) | - | Photocatalyst (20 mg), $H_2O$ (20 mL), simulated sunlight irradiation (Xe-Hg lamp 150 W, 1.5 AM filter), 35 ºC, 22 h | 185 and 78 µmol $g^{-1}$ for $H_2$ and $O_2$, respectively | [1] |
| Oxygen-plasma treated MIL-125(Ti)-$NH_2$ | - | Photocatalyst (20 mg), $H_2O$ (20 mL), 35 ºC, solar simulator (1 sun), reaction time 22 h. | ~83 and 29 µmol $g^{-1}$ for $H_2$ and $O_2$, respectively | [3] |
| UiO-66(Zr/Ce/Ti) | - | Photocatalyst (20 mg), $H_2O$ (20 mL), visible light irradiation (Hg-Xe lamp 150 W, $\lambda > 450$ nm) | 210 and 70 µmol $g^{-1}$ for $H_2$ and $O_2$, respectively | [4] |
| Liposome-based MOF | Pt-porphyrin; Ir-bipyridine | Photocatalyst solution containing [Ru(2,2′-bipyridine)$_3$]$^{2+}$, redox relays (tetrachlorobenzoquinone/tetrachlorobenzohydrosemiquinone) and $Fe^{3+}/Fe^{2+}$), $H_2O$ (20 | 836 and 418 µmol $g^{-1}$ for $H_2$ and $O_2$, respectively | [5] |

| | | | | |
|---|---|---|---|---|
| | | mL), 72 h | | |
| UiO-66-$NH_2$ | Pt, Mn | Photocatalyst (10mg), $H_2O$ (100 mL), visible light irradiation (Xe lamp 300W, λ> 400 nm) | 19.6 μmol $g^{-1}$ $h^{-1}$,and 10.1 μmol $g^{-1}$ $h^{-1}$ for $H_2$ and $O_2$, respectively | 6 |


1. P. Salcedo-Abraira, A. A. Babaryk, E. Montero-Lanzuela, O. R. Contreras-Almengor, M. Cabrero-Antonino, E. S. Grape, T. Willhammar, S. Navalón, E. Elkäim and H. García, *Adv. Mater.*, 2021, **33**, 2106627.
2. X. Qiu, Y. Zhu, X. Zhang, Y. Zhang, L. T. Menisa, C. Xia, S. Liu and Z. Tang, *Sol. RRL*, 2020, **4**, 1900449.
3. M. Cabrero-Antonino, J. Albero, C. García-Vallés, M. Álvaro, S. Navalón and H. García, *Chem. Eur. J.*, 2020, **26**, 15682-15689.
4. A. Melillo, M. Cabrero-Antonino, S. Navalón, M. Álvaro, B. Ferrer and H. Garcia, *Appl. Catal. B: Environ.*, 2020, **278**, 119345.
5. H. Hu, Z. Wang, L. Cao, L. Zeng, C. Zhang, W. Lin and C. Wang, *Nat. Chem.*, 2021, **13**, 358-366.
6. J. Zhang, T. Bai, H. Huang, M. H. Yu, X. Fan, Z. Chang and X. H. Bu, *Adv. Mater.*, 2020, **32**, 2004747.
